# Socioeconomic inequalities in HIV/AIDS prevalence in sub-Saharan African countries: evidence from the Demographic Health Surveys

**DOI:** 10.1186/1475-9276-13-18

**Published:** 2014-02-18

**Authors:** Mohammad Hajizadeh, Drissa Sia, S Jody Heymann, Arijit Nandi

**Affiliations:** 1 Institute for Health and Social Policy, McGill University, 1130 Pine Avenue West, Montreal, QC H3A 1A3, Canada; 2 Fielding School of Public Health, The University of California-Los Angeles (UCLA), 650 Charles E. Young Dr. South 16-035, Los Angeles, CA 90095-1772, US; 3 Institute for Health and Social Policy & Department of Epidemiology, Biostatistics, and Occupational Health, McGill University, 1130 Pine Avenue West, Montreal, QC H3A 1A3, Canada

**Keywords:** Socioeconomic inequality, Relative and generalized concentration indices, Decomposition analysis, HIV/AIDS, Sub-Saharan Africa

## Abstract

**Introduction:**

Extant studies universally document a positive gradient between socioeconomic status (SES) and health. A notable exception is the apparent concentration of HIV/AIDS among wealthier individuals. This paper uses data from the Demographic Health Surveys and AIDS Indicator Surveys to examine socioeconomic inequalities in HIV/AIDS prevalence in 24 sub-Saharan African (SSA) countries, the region that accounts for two-thirds of the global HIV/AIDS burden.

**Methods:**

The relative and generalized concentration indices (RC and GC) were used to quantify wealth-based socioeconomic inequalities in HIV/AIDS prevalence for the total adult population (aged 15-49), for men and women, and in urban and rural areas in each country. Further, we decomposed the RC and GC indices to identify the determinants of socioeconomic inequalities in HIV/AIDS prevalence in each country.

**Results:**

Our findings demonstrated that HIV/AIDS was concentrated among higher SES individuals in the majority of SSA countries. Swaziland and Senegal were the only countries in the region where HIV/AIDS was concentrated among individuals living in poorer households. Stratified analyses by gender showed HIV/AIDS was generally concentrated among wealthier men and women. In some countries, including Kenya, Lesotho Uganda, and Zambia, HIV/AIDS was concentrated among the poor in urban areas but among wealthier adults in rural areas. Decomposition analyses indicated that, besides wealth itself (median = 49%, interquartile range [IQR] = 90%), urban residence (median = 54%, IQR = 81%) was the most important factor contributing to the concentration of HIV/AIDS among wealthier participants in SSA countries.

**Conclusions:**

Further work is needed to understand the mechanisms explaining the concentration of HIV/AIDS among wealthier individuals and urban residents in SSA. Higher prevalence of HIV/AIDS could be indicative of better care and survival among wealthier individuals and urban adults, or reflect greater risk behaviour and incidence. Moreover, differential findings across countries suggest that effective intervention efforts for reducing the burden of HIV/AIDS in the SSA should be country specific.

## Introduction

Notwithstanding general improvement in health status worldwide, inequalities in health among different socioeconomic groups still remain one of the main challenges for public health [1]. Extant studies in both high- and low-income countries almost universally document a positive gradient between socioeconomic status (SES) and health; individuals in higher SES are in better health than lower SES individuals [2,3]. A notable exception is the apparent concentration of HIV/AIDS, one of the leading causes of death in sub-Saharan Africa (SSA) [4], among wealthier individuals [5].

Research showing socioeconomically disadvantaged groups, including women, are disproportionately affected by HIV/AIDS [6] suggest poverty is a risk factor for infection [7]. Poverty may constrain individuals’ means to negotiate safe practices, such as condom use, and avoid risky ones, including transactional sex [8]. However, growing empirical evidence suggests the prevalence of HIV/AIDS is concentrated among wealthier rather than poorer individuals in SSA. Historically, this may be a consequence of the HIV/AIDS epidemic first emerging in urban areas in SSA and then spreading to the other regions [9]. Additionally, epidemiologic evidence suggests wealthier individuals may engage in riskier behaviours, such as having multiple sexual partners, that increase the probability of HIV transmission [10,11]. Socioeconomically advantaged individuals may also have improved access to treatment for HIV/AIDS, as well as ability to adhere to treatment, prolonging survival and increasing HIV/AIDS prevalence among wealthier individuals [12].

Several studies (e.g., [5,6,13-18]) have examined whether socioeconomic status is associated with HIV/AIDS status in SSA countries. Although this work suggests HIV/AIDS is concentrated among the better-off in selected SSA countries, extant work does not report within-country summary measures of socioeconomic inequality that account for the probability of HIV/AIDS across the entire socioeconomic gradient and can be used for making cross-national comparisons. Moreover, the determinants of observed socioeconomic inequalities in HIV/AIDS have not been empirically investigated. The present study attempts to address these gaps in the literature by estimating socioeconomic inequalities in HIV/AIDS prevalence among adults aged 15-49 in 24 SSA countries using the concentration approach. Additionally, we decompose socioeconomic inequalities in HIV/AIDS prevalence to identify some of the determinants of socioeconomic inequalities in HIV/AIDS prevalence.

## Socioeconomic status and HIV/AIDS: a review of empirical studies

Studies assessing the relation between SES (as measured by education) and HIV/AIDS prevalence in SSA, a region that accounts for two thirds of the global epidemic [19], show a positive education gradient in HIV infection [5,20,21]. The concentration of HIV/AIDS among more highly educated individuals was corroborated by a meta-analysis of published studies by Hargreaves and Glynn [15], which also adjusted for gender and age. In contrast, different results were obtained in a study by Glynn and colleagues [14] in several cities in SSA; this study showed that education and HIV/AIDS were negatively associated among men in Cotonou (Benin) and women in Yaoundé (Cameroon). Using Demographic and Health Surveys (DHSs) and World Population Prospects (WPP) data from 19 SSA countries, Iorio and Santaeulalia [22] demonstrated that the association between educational attainment and HIV status is strongly related to the stage of the HIV/AIDS epidemic. During the early stage of the epidemic the association is positive, the association becomes negative as the epidemic develops, and the negative association reverses back to positive in the more advanced stage of the HIV epidemic. Other work [23] using DHSs for Lesotho, Malawi, Swaziland, and Zimbabwe also indicated that the relation between HIV infection and education varies by country.

Prior work also supports a positive relation between individual- and household-level wealth and HIV/AIDS prevalence across SSA countries [18,24-26]. However, recent studies by Fortson [5] and Asiedu et al. [23] indicated that the association between wealth and HIV infection varies by country. In addition, using information from 170 regions in sixteen SSA countries, Fox [17] demonstrated that in poorer countries/regions wealthier individuals were more likely to be HIV positive, whereas in wealthier countries/regions it was poorer individuals who had a higher probability of being infected with HIV. In summary, recent cross-national evidence suggests there is substantial heterogeneity in the magnitude and direction of the association between SES and HIV/AIDS across SSA countries.

The determinants of socioeconomic inequalities in HIV/AIDS are poorly understood. Socio-demographic factors such as age, gender, and marital status may be associated with levels of household wealth, as well as risk of HIV infection, and contribute to differences in prevalence of HIV/AIDS among SES groups. Age, for example, is associated with the accumulation of household wealth and risk of HIV infection [23,26]. Women are more biologically susceptible to HIV infection and, on average, more likely to be infected in SSA countries [6]; however, inequitable economic arrangements place women at greater risk of poverty and thus gender is unlikely to explain the concentration of HIV/AIDS among wealthier individuals. Socioeconomic factors, such as educational attainment, are positively associated with wealth and, to the extent that education is correlated with knowledge of HIV transmission [27], may help lower transmission. However, it is also possible that educational attainment is associated with riskier sexual behaviours because of differences in wealth, nature of employment or travel [28]. Behaviours themselves, which can be on the pathway between SES and risk of HIV infection, may also affect economic outcomes. For example, women with limited economic opportunities may engage in transactional sex [29], which increase risk of infection. Using DHSs from 19 SSA countries Burke and colleagues [30] showed that negative income shocks due to drought led to substantial increase in HIV/AIDS prevalence, especially for women working in agriculture. Geographic factors, such as urban residence, are positively associated with employment opportunities. In turn, urban residence might increase the probability of HIV infection via the pathways discussed, or by increasing the probability of survival conditional on infection by improving access to health services and treatment availability.

Previous work has measured the magnitude of socioeconomic inequality by comparing the prevalence of HIV/AIDS at the extremes of the socioeconomic distribution. Unlike summary measures, such as the relative and generalized concentration (RC and GC) indices, that quantify inequality across the entire SES gradient [31], this approach limits our ability to compare the magnitude of inequalities in HIV/AIDS across countries. Furthermore, the determinants of socioeconomic inequalities in HIV/AIDS are unclear. In this study, we *first* measured socioeconomic inequalities in HIV/AIDS within 24 SSA countries using the concentration approach. *Second*, since other factors (e.g., urban/rural residence) might explain the socioeconomic gradient in HIV/AIDS, we then used the decomposition property of the RC and GC indices to identify the factors that contribute to socioeconomic inequalities in HIV/AIDS prevalence.

## Methodology

### Data

The main source of data in this study is based on data collected through the Demographic Health Surveys programme in sub-Saharan African countries. The DHS programme has collected data from more than 85 low-and-middle-income countries (LMICs) around the world since 1984 [32]. DHS surveys are nationally representative cross-sectional surveys of household samples for selected LMICs [32] and collect comparable information about a wide range of topics [33]. To ensure standardisation and comparability of surveys across countries and time the DHS uses well-trained interviewers, standardized tools and measurement techniques, and a similar core set of survey questions [34,35]. Starting from 2001, the DHS programme has conducted HIV testing in the DHS or AIDS Indicator Surveys (AIS) in a number of participant countries. Availability of HIV test results data from recent DHS surveys presents a unique opportunity for population-based research about HIV/AIDS in different areas [6]. This study uses information derived from 24 DHS surveys carried out in SSA. We used the most recent survey for each country in the analysis if there was more than one available survey. Moreover, the World Bank's World Development Indicators and Global Development Finance (WDI and GDF) [36] and Worldwide Governance Indicators (WGI) [37] databases were used to obtain country-level information on socioeconomic and governance indicators.

### Measures

The primary outcome of interest in the study, HIV/AIDS infection, was determined using confirmatory HIV antibody testing. We calculated socioeconomic inequalities in HIV/AIDS using a constructed wealth index provided in all DHS. The DHS employs a method proposed by Filmer and Pritchett [38] to construct the wealth index [39] using information on household’s ownership of selected assets (e.g., bicycle and televisions), environmental conditions and housing characteristics (e.g., type of water source, sanitation facilities, materials used for housing construction).

We examined patterns of socioeconomic inequalities in HIV/AIDS prevalence according to economic, governance, social and cultural structures. The Gross Domestic Product (GDP) per capita (purchasing power parity, constant 2005 international $) was used as an indicator of country-level socioeconomic status. The World Bank’s estimated Gini index and the Country Policy and Institutional Assessment (CPIA) gender inequality rating were used as measures of state-level income and gender equality, respectively. The CPIA gender equality indicator (1 = low to 6 = high) measures the degree to which a country has installed institutions and programs to promote gender equality in access to health, the economy, education and protection under law [36]. The Worldwide Governance Indicators *viz.* voice and accountability, political stability and absence of violence, government effectiveness, regulatory quality, rule of law and control of corruption were used as measures of a country’s quality of governance (For more information about these indicators see [37]).

We collected information on demographic, socioeconomic, behavioural and ecological determinants of HIV/AIDS, based on the relevant literature (e.g., [5,6,17,18,23,25,26]). Given the literature and availability of variables across the DHSs, we used age, gender and marital status variables to control for demographic factors in our decomposition analysis. The wealth index, educational attainment, and occupation status were used to account for socioeconomic factors affecting HIV/AIDS status. As the wealth index contains negative values, similarly to previous studies (e.g. [40,41]), we normalized it to a scale of 0 to 100 points to allow the calculation of the RC and GC for household wealth. To control for sexual behaviours we measured the number of sexual partners outside marriage and the age at first sex. Finally, we included dummy variables for urban areas to control for residential characteristics. Table 1 reports the definition of all variables used in the decomposition analysis.

**Table 1:** Description of the variables

**Variables**	**Description**
**Outcome variable**	
HIV/AIDS	1 = if the individual is HIV-positive, 0 otherwise
**Demographic variables**	
Age	
*15-20*	1 = if male aged 15-19 years, 0 otherwise
*20-29*	1 = if male aged 20-29 years, 0 otherwise
*30-39*	1 = if male aged 30-39 years, 0 otherwise
*40-49 (Ref.)*	1 = if male aged 40-49 years, 0 otherwise
Gender	
*Male (Ref.)*	1 = if male, 0 otherwise
*Female*	1 = if female, 0 otherwise
Marital status	
*Married (Ref.)*	1 = if the individual is married, 0 otherwise
*Separated/divorced/widowed*	1 = if the individual is separated/divorced/widowed, 0 otherwise
*Never married*	1 = if the individual is never married, 0 otherwise
**Socioeconomic variables**	
Standard of living	
*Wealth index*	Normalized wealth score on a scale of 0-100
Education level	
*None (Ref.)*	1 = if the individual has no education, 0 otherwise
*Primary*	1 = if the individual has primary education, 0 otherwise
*Secondary and above*	1 = if the individual has secondary and above education, 0 otherwise
Occupation type	
*Agriculture (Ref.)*	1 = if the individual’s occupation is agriculture, 0 otherwise
*White-collar*	1 = if the individual is employed in occupations such as management, and office/service, 0 otherwise
*Blue-collar*	1 = if the individual is employed in manual work, 0 otherwise
*Other occupations*	1 = if the individual is employed in other occupations such as trade and domestic, 0 otherwise
*Unemployed*	1 = if the individual is unemployed, 0 otherwise
**Behavioural variables**	
Number of sex partners	Number of sex partners the individual, excluding spouse, has in last 12 months.
Age at first sex	
*Never had sex*	1 = if the individual never had sex, 0 otherwise
*<16*	1 = if the individual had sex before the age of 16, 0 otherwise
*16-17*	1 = if the individual had sex in ages 16 and 17, 0 otherwise
*18-19*	1 = if the individual had sex in ages 18 and 19, 0 otherwise
*20 and above (Ref.)*	1 = if the individual had sex after the age of 19, 0 otherwise
**Ecological variable**	
Geographical area	
*Urban*	1 = if the individual resides in urban area, 0 otherwise
*Rural (Ref.)*	1 = if the individual resides in rural area, 0 otherwise

### Statistical analysis

Our statistical analysis involves the following two steps: First, we used the relative and generalized concentration indices to quantify the degree of wealth-related inequality in HIV/AIDS prevalence in sub-Saharan countries. Second, a decomposition approach was employed to identify the factors contributing to inequality in HIV/AIDS in each country.

#### The relative and generalized concentration indices

We used the concentration index approach to measure within country inequality in HIV/AIDS prevalence. The RC index, which is based on the (standard) concentration curve, quantifies the degree of socioeconomic inequality in a health-related outcome variable of interest. The concentration curve is obtained by plotting the cumulative share of the population, ranked in ascending order of SES (i.e., household wealth), against the cumulative share of the outcome variable (i.e. HIV/AIDS). The RC index is twice the area between the line representing perfect inequality and the concentration curve. The “convenient regression” approach to compute the RC index can be formulated as follows [42]:

(1) $$2\sigma_{r}^{2}\left(\frac{y_{i}}{\mu }\right)=\alpha +\varphi r_{i}+\epsilon_{i},$$

where *y*_*i*_ is the outcome variable of interest (i.e. HIV/AIDS) for individual *i*, *μ* is the mean of the outcome variable for the whole sample, *r*_*i*_ *= i/N*, is the fractional rank of individual *i* in the distribution with *i* = 1 for the poorest and *i* = *N* for the wealthiest individual, and $\sigma_{r}^{2}$ is the variance of fractional rank. The ordinary least squares estimate of *φ* is the RC [43]. As the nature of the fractional rank variable causes a certain pattern of autocorrelation in the data, the standard error of *φ* provides an estimate of the standard error of the RC which is inaccurate. The Newey-West estimator [44] can be used to correct for autocorrelation as well as heteroskedasticity [45]. The RC index is negative if ill-health outcome is concentrated among individuals of lower SES and positive if it is concentrated among those with higher SES [45]. The value of the RC ranges from -1 and +1 with zero representing perfect equality.

Wagstaff [46] demonstrated that when the outcome variable of interest is bounded between 0 and 1, the minimum and maximum of the C are not -1 and +1 and depend on *μ*. In such cases the index can be normalized by multiplying the estimated index by 1/1 - *μ*. As the outcome variable in our study is binary, we used the normalized *RC* to quantify wealth-related inequalities in HIV/AIDS prevalence.

The RC is attractive to those who are interested in relative differences in health outcomes between different SES groups. We can also generalize the concentration curve such that it becomes sensitive to variations in *μ* and reflects absolute, rather than relative, differences in health between socioeconomic groups. The generalized concentration curve is the standard concentration curve multiplied by the *μ*. It represents the cumulative share of population, ranked based on a socioeconomic factor, against the cumulative amount of health-related variable. The generalized (absolute) concentration index (GC) is defined as twice the area between the generalized concentration curve and the diagonal (i.e. perfect equality line). The GC can be formulated as:

(2) GC=μ×RC.

The GC ranges from -*μ* to *μ*, with zero indicating “no disparity” [31].

The RC and GC do not take into account the contribution of demographics (unavoidable factors) to overall socioeconomic inequalities in health. Thus, using the direct standardisation approach [47] we corrected for differences in demographic composition and measured standardized relative and generalized concentration indices (SRC and SGC) for HIV/AIDS prevalence. The SRC and SGC demonstrate avoidable health inequality, making it more relevant for policy interventions [48].

We estimated a summary measure of socioeconomic inequality in HIV/AIDS prevalence across sampled countries by ranking countries based on their GDP per capita and estimating the RC and GC. We measured wealth-related inequalities in HIV/AIDS prevalence by gender and place of residence to examine gender and urban/rural differences in the association between socioeconomic status and HIV/AIDS prevalence. A method suggested by Altman and Bland [49] was used to assess the significance of differences in socioeconomic inequalities across gender and place of residence at the p-value = 0.05 level with 95% confidence intervals.

To examine patterns of socioeconomic inequalities in HIV/AIDS prevalence, in a framework similar to [48], we also compared the estimated RC/SRC and GC/SGC for HIV/AIDS prevalence across countries with respect to economic, governance, social and cultural structures. We first assessed socioeconomic patterns of inequality in HIV/AIDS with regard to the living standard of countries, measured by GDP per capita. Additionally, recent studies (e.g. [50]) suggest a positive association between income and gender inequalities and HIV/AIDS prevalence in SSA region. Thus, we investigated the association between Gini index and the CPIA gender equality and socioeconomic inequalities in HIV/AIDS prevalence across countries. Since some studies (e.g. [51]) suggested a negative association between governance and HIV/AIDS prevalence, we further compared the association between the RC/SRC and GC/SGC and the Worldwide Governance Indicators. Finally, we investigated the pattern of socioeconomic inequality across countries with different social and cultural characteristics by using three dummy variables for neighbouring countries: Western Africa (Burkina Faso, Cote d’Ivoire, Ghana, Guinea, Liberia, Mali, Niger, Senegal and Sierra Leone), Eastern and Central Africa (Cameroon, Congo Brazzaville, Congo Democratic Republic, Ethiopia, Kenya, Rwanda, Uganda, Sao Tome & Principe and Tanzania), Southern Africa (Lesotho, Malawi, Mozambique, Swaziland, Zambia and Zimbabwe). In all regressions we also included a survey year variable to capture the effect of temporal variation.

#### Decomposition of the relative and generalized concentration indices

A decomposition technique was employed to quantify and compare the extent to which observed determinants of HIV/AIDS, such as education, age, gender, and marital status, contributed to the socioeconomic inequality in HIV/AIDS in each country. Suppose we start with a linear regression model linking our variable of interest, *y*, to a set of *k* explanatory factors, *x*_*k*_, such as:

(3) $$y=\propto +\sum_{k}\beta_{k}x_{k}+\epsilon .$$

Wagstaff et al. [52] showed that the RC index of *y* can be decomposed into the contribution of factors which determine HIV/AIDS. They demonstrated that the RC index for *y*, can be formulated as:

(4) $$\mathrm{RC}=\sum_{k}\left(\frac{\beta_{k}\bar{x_{k}}}{\mu }\right)RC_{k}+\frac{GC_{\epsilon }}{\mu },$$

where $\bar{x_{k}}$ is the mean of *x*_*k*_, *RC*_*k*_ is the RC index for *x*_*k*_, and *GC*_*ϵ*_ is the generalized concentration index for the error term defined as $GC_{\epsilon }=\frac{2}{n}\sum_{i=1}^{n}\epsilon_{i}r_{i}$, where *r*_*i*_ is the fractional rank of the *i*th person in the relevant distribution [52]. The residual component (the error term) in equation (4) reflects the wealth-related inequality in HIV/AIDS prevalence that is not explained by systematic differences in *x*_*k*_ across wealth groups [43]. Using the Wagstaff’s correction to normalize the *RC* index yields:

(5) $$RC_{\mathrm{normalized}}=\frac{\mathrm{RC}}{1-\mu }=\frac{\sum_{k}\left(\frac{\beta_{k}\bar{x_{k}}}{\mu }\right)RC_{k}}{1-\mu }+\frac{\frac{GC_{\epsilon }}{\mu }}{1-\mu }.$$

The decomposition of the generalized concentration index can be written as:

(6) $$GC_{\mathrm{normalized}}=\frac{\mathrm{\mu RC}}{1-\mu }=\frac{\sum_{k}\beta_{k}GC_{k}}{1-\mu }+\frac{GC_{\epsilon }}{1-\mu },$$

where *GC*_*k*_ indexes the generalized concentration index for the determinant *k*. According to Equation 6 the extent of the contribution of each factor (*x*_*k*_) to the *GC* in HIV/AIDS prevalence depends on the *β*_*k*_ and *GC*_*k*_. A factor that influences the probability of HIV/AIDS and is distributed unequally by wealth can contribute to socioeconomic inequality in HIV/AIDS prevalence.

A limitation of this decomposition approach is that it only works with linear models. Thus, although it is preferable to use a non-linear estimator in our application because our outcome is binary variable, we employed a linear probability model (LPM) in the analysis. Some approaches have been proposed to address the issue [53]. These methods, however, lead to other problems and restrictions, such that they are not explicitly preferable to using LPM [54].

## Results

### Descriptive results

Table 2 presents the sample size, GDP per capita (constant 2005 international $), and overall and gender-specific HIV/AIDS prevalence for each county. HIV/AIDS prevalence ranged from less than 1 per cent in Senegal and Niger to greater than 20 per cent in Swaziland and Lesotho. Gender differentials in HIV/AIDS varied widely across countries, with prevalence higher for women in all countries except Sao Tom & Principe. With the exception of Niger, Sao Tom & Principe and Senegal, prevalence of HIV/AIDS was higher in urban compared to rural areas.

**Table 2:** Survey year, sample size, GDP per capita and HIV/AIDS prevalence in SSA countries

**Name of country**	**Country code**	**Survey year**	**Sample size (total)**	**GDP per capita**	**HIV/AIDS prevalence**				
					**Total**	**Male**	**Female**	**Urban**	**Rural**
Burkina Faso	BF	2010	15380	283	1.02	0.84	1.17	2.07	0.62
Cameroon	CM	2011	14198	666	4.25	2.89	5.57	4.67	3.76
Congo Brazzaville	CG	2009	12109	101	3.16	2.06	4.12	3.35	2.84
Congo Democratic Republic	CD	2007	8936	98	1.27	0.92	1.62	1.86	0.80
Cote d’Ivoire	CI	2005	8464	578	4.74	3.11	6.21	5.45	4.10
Ethiopia	ET	2011	28503	230	1.43	0.98	1.86	4.16	0.62
Ghana	GH	2003	9554	276	2.20	1.63	2.71	2.30	2.12
Guinea	GN	2005	6767	385	1.54	1.10	1.89	2.65	0.95
Kenya	KE	2008/2009	6906	457	6.36	4.55	26.43	7.25	6.07
Lesotho	LS	2009/2010	6924	485	22.97	18.45	26.73	26.83	21.27
Liberia	LR	2005	11688	187	1.60	1.23	1.91	2.54	0.94
Malawi	MW	2010	13905	181	10.67	8.39	12.88	17.74	8.88
Mali	ML	2006	8629	255	1.34	1.11	1.54	1.73	1.12
Mozambique	MZ	2009	10305	368	11.11	9.04	12.67	15.52	8.95
Niger	NE	2006	7673	172	0.71	0.71	0.71	1.46	0.50
Rwanda	RW	2010	13248	353	3.09	2.41	3.71	7.03	2.37
Sao Tome & Principe	ST	2008/2009	4710	1874	1.54	1.79	1.29	0.88	2.26
Senegal	SN	2011	9917	560	0.68	0.51	0.83	0.63	0.74
Sierra Leone	SL	2008	6455	259	1.47	1.16	1.73	2.40	0.94
Swaziland	SZ	2006/2007	8186	1745	25.88	19.70	31.15	31.43	23.79
Tanzania	TZ	2007/2008	15044	420	5.67	4.56	6.61	8.73	4.69
Uganda	UG	2011	10599	393	7.28	6.11	8.21	8.85	6.89
Zambia	ZM	2007	10874	385	14.21	12.29	16.09	19.51	10.27
Zimbabwe	ZW	2010/2011	13897	335	15.32	12.66	17.71	16.84	14.65
Total†			262871	328	4.51	3.51	6.96	5.90	3.90

†These values are weighted averages. We applied total number of adults aged 15-49 years in each country (calculated from the United Nations World Population Database) as a weight in the calculation.

**Figure 1:**
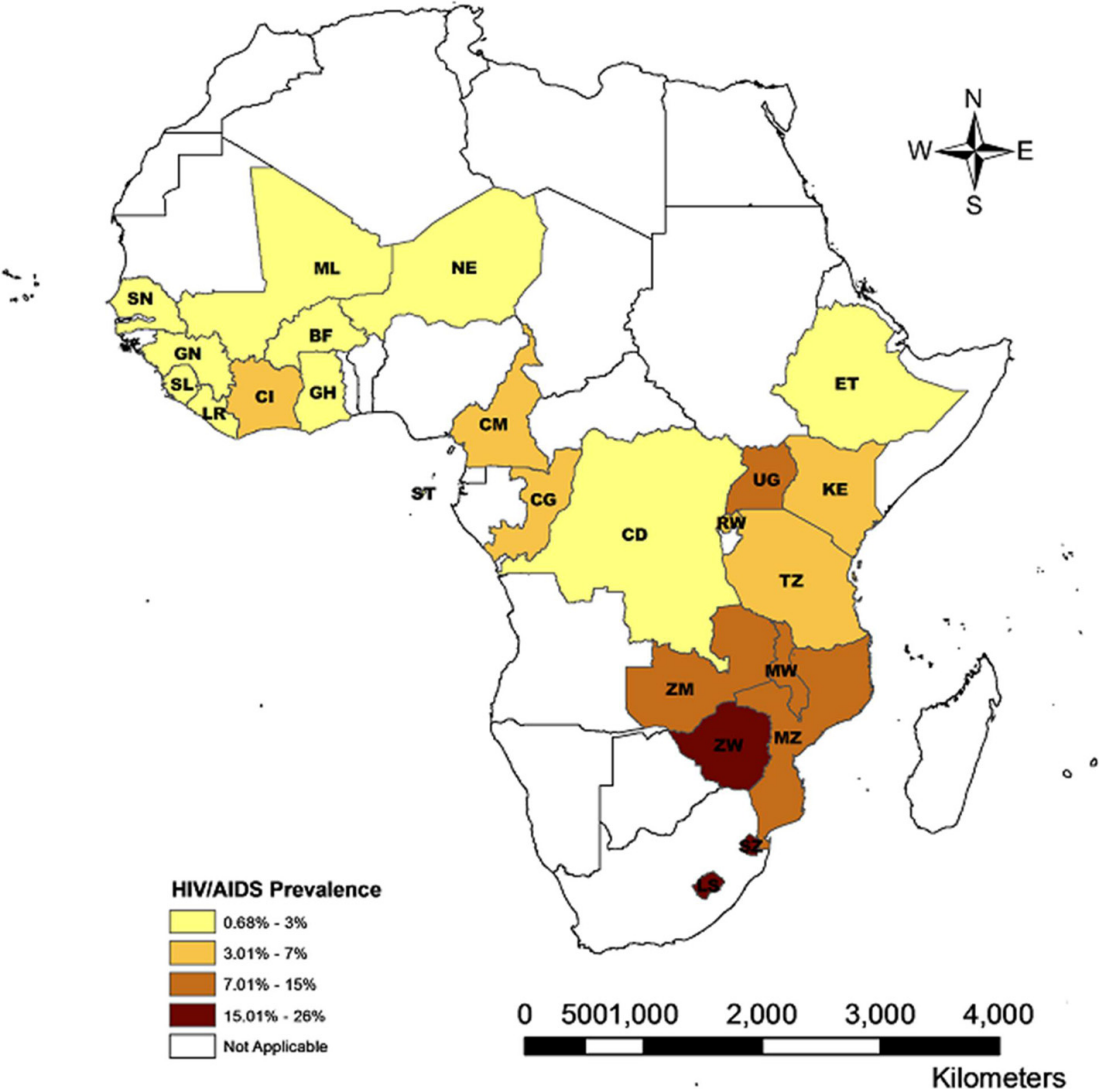
Prevalence of HIV/AIDS in SSA region.

**Figure 2:**
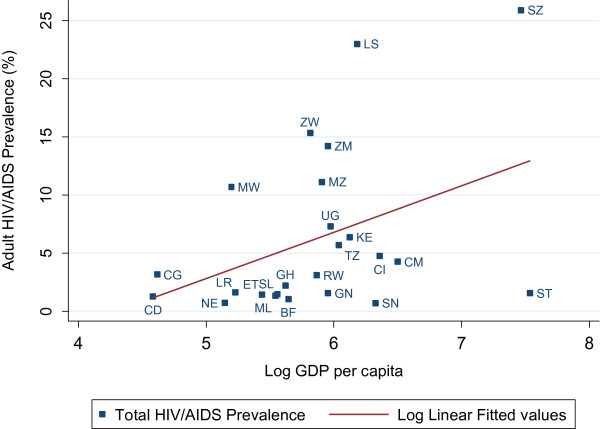
Cross-country correlation between Adult HIV/AIDS prevalence and log GDP per capita in SSA region.

As illustrated in Figure 1, there were geographic differences in HIV/AIDS levels across sub-Saharan Africa. HIV prevalence was higher in countries located in south-eastern SSA, including Swaziland, Lesotho, Zimbabwe, Zambia, Mozambique and Malawi. The descriptive statistics suggested a strong positive cross-country correlation (r(22) = 0.399, p = 0.054) between (log) per capita GDP and HIV/AIDS prevalence (See Figure 2).

### Socioeconomic inequality in HIV/AIDS

Table 3 reports the relative and generalized concentration indices for HIV/AIDS prevalence for 24 sub-Saharan countries. Using aggregate data to estimate socioeconomic inequalities in HIV/AIDS prevalence across sampled countries demonstrated that wealthier countries (based on GDP rank) in the SSA region had a greater prevalence of HIV/AIDS than their poorer counterparts (RC = 0.213 and GC = 0.96). Results stratified by gender showed that the positive association between country-level wealth and prevalence of HIV/AIDS was more pronounced for women (RC = 0.322, GC = 2.41) than for men (RC = 0.206, GC = 0.725). However, female-male differences in the RC and GC were not statistically different at the 95% confidence level (see Table 3). Results also suggested that the association between country-level wealth and HIV/AIDS was stronger among rural residents than their urban counterparts.

**Table 3:** Relative and generalized concentration indices for HIV/AIDS prevalence in SSA countries

**Country**	**Relative concentration indices**													
	**Total**		**Male**		**Female**		**RC**_**male**_**-RC**_**female**_	**SRC**_**male**_**-SRC**_**female**_	**Urban**		**Rural**		**RC**_**urban**_**-RC**_**rural**_	**SRC**_**urban**_**-SRC**_**rural**_
	**RC**	**SRC**	**RC**	**SRC**	**RC**	**SRC**			**RC**	**SRC**	**RC**	**SRC**		
BF	**0.269**	**0.298**	**0.248**	**0.009**	**0.284**	**0.324**	-0.036	**-0.315**	0.047	0.056	0.039	0.055	0.008	0.001
CM	**0.12**	**0.138**	**0.098**	**0.015**	**0.138**	**0.152**	-0.04	**-0.137**	0.059	0.06	**0.212**	**0.223**	**-0.153**	**-0.163**
CG	0.03	0.058	-0.097	-0.003	**0.09**	**0.104**	**-0.187**	**-0.107**	-0.033	-0.022	0.09	0.111	-0.123	-0.133
CD	**0.211**	**0.219**	**0.374**	**0.015**	0.125	0.153	**0.249**	-0.138	0.011	0.025	0.177	0.174	-0.166	-0.149
CI	**0.117**	**0.139**	-0.008	0.002	**0.166**	**0.193**	**-0.174**	**-0.191**	0.01	0.035	**0.145**	**0.158**	-0.135	-0.123
ET	**0.507**	**0.523**	**0.507**	**0.023**	**0.501**	**0.518**	0.006	**-0.495**	0.068	0.054	**0.266**	**0.288**	**-0.198**	**-0.234**
GH	0.038	0.052	0.013	0.002	0.044	0.065	-0.031	-0.063	-0.09	-0.1	**0.135**	**0.145**	**-0.225**	**-0.245**
GN	**0.167**	**0.209**	-0.019	0.001	**0.268**	**0.297**	**-0.287**	**-0.296**	**-0.149**	-0.117	-0.105	-0.098	-0.044	-0.019
KE	**0.07**	0.06	0.073	0.009	**0.039**	0.052	0.034	-0.043	**-0.148**	**-0.16**	**0.086**	0.083	**-0.234**	**-0.243**
LS	**0.03**	0.019	0.023	0.025	**0.026**	0.01	-0.003	0.015	-0.083	**-0.091**	**0.031**	**0.043**	**-0.114**	**-0.134**
LR	**0.244**	**0.262**	**0.276**	**0.014**	**0.226**	**0.239**	0.05	**-0.225**	0.032	0.044	0.102	0.116	-0.07	-0.072
MW	**0.143**	**0.154**	**0.127**	**0.048**	**0.155**	**0.159**	-0.028	**-0.111**	-0.012	-0.002	**0.084**	**0.087**	**-0.096**	**-0.089**
ML	0.097	0.113	0.185	0.01	0.044	0.063	0.141	-0.053	0.136	0.161	-0.058	-0.062	0.194	0.223
MZ	**0.188**	**0.211**	**0.188**	**0.085**	**0.192**	**0.203**	-0.004	**-0.118**	0.009	0.023	**0.176**	**0.189**	**-0.167**	**-0.166**
NE	**0.228**	**0.255**	0.217	0.008	**0.237**	**0.257**	-0.02	**-0.249**	-0.029	-0.013	0.028	0.026	-0.057	-0.039
RW	**0.128**	**0.15**	**0.107**	**0.016**	**0.148**	**0.161**	-0.041	**-0.145**	-0.025	-0.001	-0.019	0	-0.006	-0.001
ST	-0.063	-0.047	-0.03	0	-0.105	-0.096	0.075	0.096	-0.062	-0.044	0.025	0.035	-0.087	-0.079
SN	**-0.177**	**-0.165**	-0.215	-0.005	-0.151	-0.148	-0.064	0.143	-0.122	-0.145	**-0.248**	**-0.236**	0.126	0.091
SL	**0.255**	**0.273**	**0.41**	**0.019**	0.163	**0.169**	0.247	-0.15	0.078	0.096	0.129	0.133	-0.051	-0.037
SZ	-0.005	**-0.027**	0.011	**-0.052**	-0.01	**-0.021**	0.021	-0.031	**-0.069**	**-0.07**	**-0.033**	**-0.04**	**-0.036**	-0.03
TZ	**0.102**	**0.108**	**0.093**	**0.018**	**0.104**	**0.111**	-0.011	**-0.093**	-0.023	-0.015	-0.011	-0.004	-0.012	-0.011
UG	**0.051**	**0.064**	0.021	0.011	**0.067**	**0.08**	-0.046	**-0.069**	**-0.11**	**-0.095**	**0.042**	**0.05**	**-0.152**	**-0.145**
ZM	**0.15**	**0.177**	**0.125**	**0.076**	**0.167**	**0.187**	-0.042	**-0.111**	**-0.04**	-0.027	**0.12**	**0.133**	**-0.16**	**-0.16**
ZW	-0.017	-0.008	-0.035	-0.015	-0.002	0.006	-0.033	-0.021	**-0.072**	**-0.07**	**-0.044**	**-0.031**	-0.028	-0.039
Total†	**0.213**	-	**0.206**	-	**0.322**	**-**	-0.116		0.162	-	**0.253**	-	-0.091	
	**Generalized concentration indices**													
	**Total**		**Male**		**Female**		**GC**_**male**_**-GC**_**female**_	**SGC**_**male**_**-SGC**_**female**_	**Urban**		**Rural**		**GC**_**urban**_**-GC**_**rural**_	**SGC**_**urban**_**-SGC**_**rural**_
	**CG**	**SGC**	**GC**	**SGC**	**GC**	**SGC**			**GC**	**SGC**	**GC**	**SGC**		
BF	**0.274**	**0.304**	**0.208**	**0.008**	**0.333**	**0.38**	-0.125	**-0.372**	0.098	0.118	0.024	0.033	0.074	0.085
CM	**0.51**	**0.587**	**0.283**	**0.044**	**0.771**	**0.846**	**-0.488**	**-0.802**	0.276	0.28	**0.797**	**0.847**	**-0.521**	**-0.567**
CG	0.096	0.184	-0.2	-0.007	**0.369**	**0.429**	**-0.569**	**-0.436**	-0.108	-0.073	0.256	0.31	-0.364	-0.383
CD	**0.269**	**0.279**	**0.343**	**0.014**	0.202	0.248	0.141	-0.234	0.02	0.048	0.141	0.139	-0.121	-0.091
CI	**0.553**	**0.659**	-0.025	0.006	**1.029**	**1.202**	-1.054	**-1.196**	0.056	0.189	**0.596**	**0.648**	-0.54	-0.459
ET	**0.726**	**0.749**	**0.495**	**0.022**	**0.932**	**0.964**	**-0.437**	**-0.942**	0.285	0.225	**0.164**	**0.173**	0.121	0.052
GH	0.085	0.114	0.022	0.004	0.118	0.176	-0.096	-0.172	-0.208	-0.23	**0.286**	**0.305**	**-0.494**	**-0.535**
GN	**0.257**	**0.322**	-0.021	0.001	**0.506**	**0.561**	**-0.527**	**-0.56**	**-0.402**	-0.315	-0.1	-0.098	-0.302	-0.217
KE	**0.447**	0.379	0.334	0.042	**1.03**	1.375	-0.696	-1.333	**-1.08**	**-1.168**	**0.519**	0.507	**-1.599**	**-1.675**
LS	**0.685**	0.426	0.425	0.463	**0.685**	0.273	-0.26	0.19	**-2.229**	**-2.445**	**0.649**	**0.926**	**-2.878**	**-3.371**
LR	**0.39**	**0.418**	**0.34**	**0.017**	**0.431**	**0.456**	-0.091	**-0.439**	0.081	0.11	0.095	0.104	-0.014	0.006
MW	**1.524**	**1.643**	**1.063**	**0.4**	**1.998**	**2.054**	**-0.935**	**-1.654**	-0.207	-0.033	**0.747**	**0.774**	-0.954	-0.807
ML	0.13	0.151	0.205	0.011	0.067	0.097	0.138	-0.086	0.232	0.274	-0.065	-0.068	0.297	0.342
MZ	**2.094**	**2.342**	**1.702**	**0.764**	**2.428**	**2.577**	-0.726	**-1.813**	0.143	0.364	**1.575**	**1.684**	**-1.432**	**-1.32**
NE	**0.162**	**0.181**	0.155	0.006	**0.168**	**0.182**	-0.013	**-0.176**	-0.044	-0.019	0.014	0.013	-0.058	-0.032
RW	**0.394**	**0.462**	**0.257**	**0.038**	**0.549**	**0.598**	-0.292	**-0.56**	-0.173	-0.01	-0.044	0	-0.129	-0.01
ST	-0.098	-0.073	-0.055	0.000	-0.136	-0.124	0.081	0.124	-0.056	-0.04	0.056	0.081	-0.112	-0.121
SN	**-0.121**	**-0.112**	-0.11	-0.003	-0.125	-0.123	0.015	0.12	-0.073	-0.087	**-0.183**	**-0.165**	0.11	0.078
SL	**0.373**	**0.4**	**0.477**	**0.022**	0.282	**0.292**	0.195	-0.27	0.188	0.23	0.121	0.12	0.067	0.11
SZ	-0.141	**-0.705**	0.219	**-1.016**	-0.319	**-0.648**	0.538	-0.368	**-2.174**	**-2.202**	**-0.79**	**-0.95**	**-1.384**	**-1.252**
TZ	**0.578**	**0.615**	**0.425**	**0.083**	**0.69**	**0.731**	-0.265	**-0.648**	-0.2	-0.127	-0.053	-0.021	-0.147	-0.106
UG	**0.37**	**0.469**	0.129	0.065	**0.553**	**0.653**	**-0.424**	**-0.588**	**-0.982**	**-0.845**	**0.287**	**0.343**	**-1.269**	**-1.188**
ZM	**2.13**	**2.511**	**1.531**	**0.935**	**2.689**	**3.016**	**-1.158**	**-2.081**	**-0.782**	-0.522	**1.233**	**1.37**	**-2.015**	**-1.892**
ZW	-0.256	-0.129	-0.444	-0.188	-0.032	0.105	-0.412	-0.293	**-1.212**	**-1.17**	**-0.645**	**-0.455**	-0.567	**-0.715**
Total†	**0.96**	-	**0.725**	**-**	**2.241**	**-**	-1.516		0.957	**-**	**0.977**	**-**	-0.02	

***Note*:** Bold font indicates statistically significantly different from zero at the five per cent level; GC and SGC is multiplied by 100 for ease of interpretation.

†We calculated the overall RC and GC by ranking countries based on their GDP per capita. Also, we applied total number of adults (15-59 years) during the study period for each country as a weight in the calculation.

Within countries HIV/AIDS prevalence was concentrated among the socioeconomically advantaged, based on household wealth, in the majority of SSA countries. Swaziland and Senegal were the only countries with negative RC/SRC and/or GC/SGC. Results of the RC/SRC suggested that the relative concentration of HIV/AIDS prevalence among the better-off was higher in countries such as Ethiopia, Burkina Faso, Sierra Leon, Liberia, Niger, and Congo Democratic Republic, whereas the absolute concentration of HIV/AIDS among wealthier individuals was greatest in Zambia, Mozambique, Malawi, Ethiopia, Lesotho, and Tanzania. Although, the calculated RC/SRC and/or GC/SGC generally suggested similar patterns of socioeconomic inequality in HIV/AIDS within countries for men and women, the concentration of HIV/AIDS prevalence among individuals from wealthier households was greater for women than for men (see statistically significant negative values of the difference in socioeconomic inequalities indices for men and women in Table 3). Results also showed that HIV/AIDS was more prevalent among the poor in urban areas in countries such as Uganda, Kenya, Zimbabwe and Swaziland. In rural areas, however, HIV/AIDS was more prevalent among wealthier individuals in most countries.

**Figure 3:**
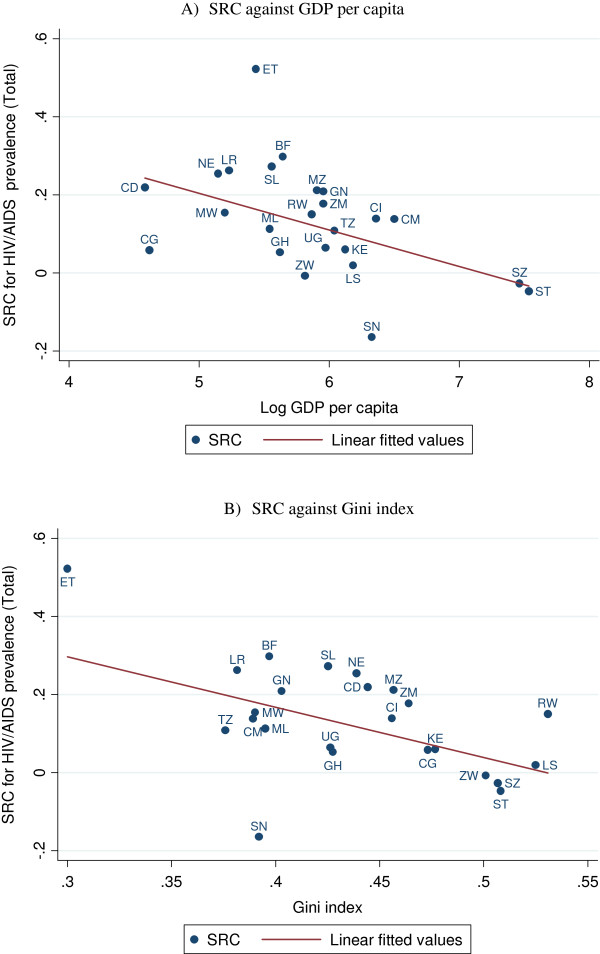
**Cross-country correlations between the SRC for HIV/AIDS prevalence (total) and log GDP per capita and Gini index in SSA region. A)** SRC against GDP per capita. **B)** SRC against Gini index.

Table 4 reports multivariate regression results using the RC, SRC, GC and SGC as dependent variables. As illustrated by Figure 3, there was a statistically significant negative relation between the living standard of countries, measured by GDP per capita, and the RC/SRC for HIV/AIDS, indicating that HIV/AIDS was less concentrated among the better-off in wealthier countries. Similarly, the Gini index was negatively associated with the RC and SRC. There was also a positive association between countries in the region of Southern Africa and the GC, suggesting that absolute socioeconomic inequality for HIV/AIDS was greater in these countries relative to countries in other regions. Country-level gender inequality and governance indicators were not consistently associated with levels of inequality in HIV/AIDS.

**Table 4:** Aggregate multivariate regressions

**Independent variables**	**GDP/Cap (Log)**	**Gini index**	**Gender equality†**	**Governance indicators**						**Western Africa**	**Eastern and Central Africa**	**Southern Africa**
**Dependent variables**				**Control of corruption**	**Government effectiveness**	**Political stability**	**Regulatory quality**	**Rule of law**	**Voice and accountability**			
**Total**												
**RC**	**-0.093**	**-1.298**	-0.057	-0.035	-0.032	-0.046	-0.040	-0.022	-0.043	0.023	0.028	-0.050
**SRC**	**-0.100**	**-1.376**	-0.057	-0.041	-0.036	-0.046	-0.043	-0.024	-0.045	0.021	0.042	-0.061
**GC**	-0.126	-1.119	0.242	0.224	0.319	0.194	0.281	0.259	0.264	-0.264	-0.456	**0.703**
**SGC**	-0.220	-1.816	0.224	0.124	0.285	0.191	0.260	0.229	0.302	-0.262	-0.420	0.667
**Male**												
**RC**	**-0.107**	**-1.327**	-0.086	-0.026	-0.059	-0.039	-0.045	-0.012	-0.001	0.033	0.017	-0.052
**SRC**	-0.012	-0.077	0.011	0.008	0.014	0.009	0.012	0.013	0.019	-0.008	-0.010	0.018
**GC**	-0.067	-0.902	0.208	0.286	0.298	0.220	0.296	0.294	0.298	-0.216	-0.371	**0.572**
**SGC**	-0.144	-0.368	0.172	0.090	0.177	0.122	0.138	0.155	**0.271**	-0.117	-0.073	0.194
**Female**												
**RC**	**-0.089**	**-1.312**	-0.086	-0.036	-0.018	-0.049	-0.038	-0.025	-0.069	0.009	0.037	-0.044
**SRC**	**-0.095**	**-1.387**	0.011	-0.045	-0.024	-0.054	-0.038	-0.028	-0.073	0.013	0.046	-0.057
**GC**	-0.157	-1.157	0.208	0.100	0.324	0.137	0.297	0.180	0.214	-0.241	-0.588	**0.798**
**SGC**	-0.210	-1.642	0.172	-0.054	0.274	0.082	0.309	0.105	0.213	-0.166	-0.592	0.720
**Urban**												
**RC**	-0.033	**-0.549**	-0.046	-0.005	-0.035	0.004	-0.025	0.001	0.006	-0.009	0.039	-0.026
**SRC**	-0.035	-0.511	-0.047	-0.007	-0.043	0.004	-0.029	-0.004	0.002	-0.005	0.038	-0.029
**GC**	-0.401	**-7.420**	-0.348	-0.353	-0.182	-0.113	-0.034	-0.044	0.117	0.291	**0.717**	**-0.970**
**SGC**	-0.418	**-7.456**	-0.335	-0.363	-0.190	-0.100	-0.015	-0.043	0.107	0.274	0.691	**-0.927**
**Rural**												
**RC**	-0.045	-0.388	-0.077	-0.057	-0.045	-0.035	-0.040	-0.034	-0.023	0.078	-0.091	-0.001
**SRC**	-0.047	-0.400	-0.076	-0.056	-0.044	-0.035	-0.043	-0.034	-0.025	0.078	-0.089	-0.003
**GC**	-0.099	-0.309	0.102	0.039	0.220	0.118	0.287	0.186	0.296	-0.040	-0.273	0.293
**SGC**	-0.113	0.127	0.129	0.051	0.222	0.134	0.254	0.177	0.316	-0.083	-0.298	0.362

***Note*:** All multivariate regressions included an independent variable and survey year variable; Bold font indicates statistically significantly different from zero at the five per cent level.

†Data was not available for Swaziland.

### Determinants of socioeconomic inequalities in HIV/AIDS

Table 5 presents beta coefficients in the decomposition regression, Equation 3, in 24 SSA countries. The coefficients indicate the effect of each explanatory factor on the probability of HIV/AIDS in each country.

**Table 5:** Coefficient results (full sample)

	**Age-Gender**				**Marital status**		**Living standard**	**Education**		**Occupation**				**Sexual Behaviours**					**Urban residence**
**Country**	**15-20**	**21-29**	**31-39**	**Female**	**Never married**	**Separated/divorced/widowed**	**Wealth index**	**Primary**	**Secondary and above**	**White collar**	**Blue collar**	**Other**	**Unemployed**	**Number of sex partners**	**Never had sex**	**<16**	**16-17**	**18-19**	**Urban**
**BF**	-0.0147†	-0.0104†	-0.0008	0.0053‡	-0.0054	0.0333‡	0.000	0.0029	0.0031	0.0006	-0.0042	0.0045	-0.0039	0.0049	0.0077*	-0.0147†	-0.0104†	-0.0008	0.0053‡
**CM**	-0.0284†	-0.0142‡	0.015‡	0.0241†	-0.0109*	0.0745†	0.0004†	0.0299†	0.0316†	-0.0034	-0.0011	0.0085	-0.0039	-0.0002	-0.0042	-0.0284†	-0.0142‡	0.015‡	0.0241†
**CG**	-0.0543†	-0.0333†	-0.0201‡	0.0231†	0.0132*	0.0265†	0.0001	0.0079	0.0115	--	--	--	--	0.0006	0.0107	-0.0543†	-0.0333†	-0.0201‡	0.0231†
**CD**	-0.0012	-0.0059	-0.0008	0.0053	-0.0027	0.0122	0000	0.0081*	0.0051	0.0071*	-0.0023	--	0.0031	0.0014	-0.0005	-0.0012	-0.0059	-0.0008	0.0053
**CI**	-0.055†	-0.0263‡	0.0145	0.0276†	0.0095	0.0647†	0.0004	0.0075	0.0024	0.008	0.0064	0.0201*	0.019	-0.0046‡	-0.0315*	-0.055†	-0.0263‡	0.0145	0.0276†
**ET**	-0.0085†	-0.0029	0.01†	0.0019	-0.0106‡	0.0481†	0.0006†	0.0086†	0.0004	-0.0043	0.0212†	0.0032	-0.0005	0.0053	0.0048	-0.0085†	-0.0029	0.01†	0.0019
**GH**	-0.0201†	-0.012‡	0.0068	0.0058*	-0.0074	0.0197‡	-0.0001	0.0162†	0.011‡	0.0094	-0.0029	0.0125*	0.006	0.001	-0.0012	-0.0201†	-0.012‡	0.0068	0.0058*
**GN**	-0.0142‡	-0.0118‡	-0.0055	0.0117†	-0.0049	0.0318	-0.0003‡	0.004	0.0107	0.0151	0.0036	0.0131‡	-0.0042	0.0069*	0.0039	-0.0142‡	-0.0118‡	-0.0055	0.0117†
**KE**	-0.0042	0.0032	0.0162	0.0265†	-0.0147	0.1884†	0.0006*	0.0325‡	0.0051	0.0274*	-0.0022	0.0137	-0.0121	0	-0.0232	-0.0042	0.0032	0.0162	0.0265†
**LS**	-0.1687†	-0.0346*	0.1048†	0.0646†	-0.0428‡	0.2234†	0.000	0.008	-0.005	-0.0167	0.0628†	0.0883†	0.0051	0.0194	-0.0217	-0.1687†	-0.0346*	0.1048†	0.0646†
**LR**	-0.0148*	-0.007	0.0001	0.0093‡	0.007	0.0123*	0.000	0.0047	0.0059	0.0152‡	0.0123	0.013‡	0.0169†	-0.0028*	-0.0103	-0.0148*	-0.007	0.0001	0.0093‡
**MW**	-0.1041†	-0.09†	0.0068	0.028†	-0.047†	0.1583†	0.0007‡	0.0152	0.0122	0.0956†	0.0178*	0.0299†	-0.0006	0.0128*	0.0478†	-0.1041†	-0.09†	0.0068	0.028†
**ML**	-0.0124‡	-0.0057	0.0011	0.0044	-0.0027	0.0147	0.0002	0.0042	-0.0011	0.0189	-0.0069	0.004	0.0001	0.0028	0.0062	-0.0124‡	-0.0057	0.0011	0.0044
**MZ**	-0.0051	0.0358†	0.048†	0.0303†	-0.0497†	0.1285†	0.0015†	0.0266†	-0.0063	-0.0456	-0.0203	-0.07	-0.0921‡	0.0062	0.0097	-0.0051	0.0358†	0.048†	0.0303†
**NE**	-0.0036	0.0021	0.0098†	0.0013	-0.0052	0.0418†	0.000	0.0007	-0.0042	0.0099	-0.0002	-0.001	-0.0027	0.0114*	0.007	-0.0036	0.0021	0.0098†	0.0013
**RW**	-0.0311†	-0.0264†	-0.0063	0.0104†	-0.0065	0.0676†	0.0001	0.0065	0.007	0.0235‡	0.0154†	0.0135*	0.0124‡	0.0061	-0.0126‡	-0.0311†	-0.0264†	-0.0063	0.0104†
**ST**	-0.0155*	-0.01	0.0033	-0.0016	-0.003	0.0095	0.0001	-0.021	-0.0181	-0.014	-0.0175*	-0.0193*	-0.0121	0.0003	0.0151	-0.0155*	-0.01	0.0033	-0.0016
**SN**	-0.0156†	-0.0115†	-0.0059	0.0002	0.0041	0.0182‡	-0.0001‡	-0.0012	-0.005‡	-0.0013	-0.0074†	-0.0008	0.0001	0.001	0.0015	-0.0156†	-0.0115†	-0.0059	0.0002
**SL**	-0.0121	-0.0038	-0.0008	0.0063	0.0067	0.0134	0.0004*	-0.0001	-0.0043	-0.004	0.0076	0.0014	-0.0032	0.0028	-0.0042	-0.0121	-0.0038	-0.0008	0.0063
**SZ**	-0.0482‡	0.109†	0.1685†	0.0852†	-0.0691†	0.1931†	-0.001†	-0.012	-0.0507‡	-0.0501‡	0.0004	0.0336	-0.0416‡	0.0468†	-0.0715†	-0.0482‡	0.109†	0.1685†	0.0852†
**TZ**	-0.036†	-0.0066	0.0279†	0.0019	-0.0086	0.092†	0.0002	0.0046	-0.0117	-0.0034	-0.0109	0.0261‡	-0.0009	-0.0011	0.0121	-0.036†	-0.0066	0.0279†	0.0019
**UG**	-0.0409†	-0.0183†	0.0223†	0.0049	-0.0112	0.1087†	0.0003*	0.0171‡	-0.0019	0.025†	0.0226†	0.0313†	0.0129‡	0.0014	0.0104	-0.0409†	-0.0183†	0.0223†	0.0049
**ZM**	-0.0556†	-0.0294‡	0.0545†	0.02‡	-0.0688†	0.1928†	0.0008†	0.0433†	0.0405†	0.0704†	0.0457†	0.0509†	0.0334†	0.0236†	-0.0039	-0.0556†	-0.0294‡	0.0545†	0.02‡
**ZW**	-0.1666†	-0.1152†	0.0091	0.0205†	-0.013	0.2179†	-0.001†	0.0925†	0.0991†	-0.0121	0.0062	0.0014	0.0047	-0.0009	0.0104	-0.1666†	-0.1152†	0.0091	0.0205†

***Note:*** Significant levels are †, ‡, * for 1, 5 and 10% respectively.

Among socio-demographic characteristics, younger age was associated with lower HIV/AIDS prevalence in the majority of SSA countries. Women had a greater burden of HIV/AIDS than men in all countries. Additionally, being separated, divorced and widowed was consistently associated with higher probability of being HIV positive compared to married individuals, whereas those who never married were at lower risk of being HIV positive than married individuals.

With respect to SES, there was a positive association between wealth and HIV/AIDS in most countries, including Cameroon, Ethiopia, Lesotho, Malawi, Mozambique and Zambia. A few countries (i.e., Guinea, Senegal, Swaziland, and Zimbabwe) showed the opposite pattern. Greater educational attainment (i.e. secondary and above) was associated with higher probability of being HIV positive in Cameroon, Ghana, Zambia, and Zimbabwe; this association was negative in countries such as Senegal, Swaziland, and Tanzania. Compared to agriculture workers, individuals with other occupations had higher prevalence of HIV/AIDS in Liberia, Malawi, Rwanda, Uganda, and Zambia, but lower prevalence in Sao Tome & Principe and Swaziland.

Multiple partners and early sexual activity were positively associated with HIV/AIDS in some countries. For example, number of sexual partners was positively associated with HIV/AIDS in Ghana, Malawi, Niger, Swaziland, and Zimbabwe. Additionally, younger age of first sexual experience was associated with the probability of being HIV positive in Ghana, Malawi, Sao Tome & Principe, Senegal, Sierra Leone, Tanzania, Uganda, Zambia and Zimbabwe. Results also showed that residing in urban areas was associated with higher probability of being HIV positive in most SSA countries.

Table 6 reports the relative and generalized concentration indices, *RC*_*k*_ and *GC*_*k*_, for all explanatory variables included in the decomposition analysis. A positive value of the *RC*_*k*_ and *GC*_*k*_ indicates that variable *x*_*k*_ is concentrated among socioeconomically advantaged individuals, and *vice versa*. Results of the *RC*_*k*_ and *GC*_*k*_ suggest that individuals who were never married, reported completion of secondary school, worked in white collar occupations, had sexual partners outside their marriage and resided in urban areas were relatively wealthier in all countries studied.

**Table 6:** Concentration index and generalized concentration index of independent variables (full sample)

		**Age-Gender**				**Marital status**		**Living standard**	**Education**		**Occupation**				**Sexual behaviours**					**Urban residence**
**Country**		**15-20**	**21-29**	**31-39**	**Female**	**Never married**	**Separated/ divorced/widowed**	**Wealth Index**	**Primary**	**Secondary and above**	**White collar**	**Blue collar**	**Other**	**Unemployed**	**Number of Sex Partners**	**Never had sex**	**<16**	**16-17**	**18-19**	**Urban**
**BF**	*RC*	0.022	0.058	0.002	0.000	0.148	0.061	0.505	0.225	0.638	0.724	0.242	0.309	0.215	0.350	0.054	-0.123	-0.018	0.046	0.637
	*GC*	0.004	0.019	0.001	0.000	0.035	0.002	7.269	0.037	0.083	0.027	0.022	0.053	0.030	0.045	0.008	-0.018	-0.004	0.009	0.170
**CM**	*RC*	0.011	0.053	-0.007	-0.016	0.129	0.021	0.346	-0.195	0.288	0.487	0.159	0.152	0.160	0.350	0.054	-0.123	-0.018	0.046	0.395
	*GC*	0.003	0.019	-0.001	-0.008	0.047	0.001	11.829	-0.065	0.149	0.032	0.033	0.030	0.037	0.182	0.008	-0.033	-0.004	0.008	0.214
**CG**	*RC*	0.036	0.052	-0.011	-0.011	0.088	-0.078	0.320	-0.306	0.123	--	--	--	--	0.021	0.119	-0.092	0.061	0.081	0.330
	*GC*	0.007	0.019	-0.003	-0.006	0.029	-0.009	12.765	-0.072	0.089	--	--	--	--	0.021	0.119	-0.092	0.061	0.081	0.208
**CD**	*RC*	0.085	0.008	-0.009	-0.001	0.161	-0.098	0.465	-0.201	0.244	0.342	0.253	--	0.178	0.038	0.166	-0.112	0.016	0.028	0.438
	*GC*	0.017	0.003	-0.002	-0.001	0.050	-0.007	12.477	-0.069	0.125	0.088	0.027	--	0.053	0.058	0.054	-0.053	0.007	0.011	0.195
**CI**	*RC*	0.057	0.029	-0.030	0.008	0.140	0.001	0.352	0.002	0.316	0.354	0.280	0.108	0.129	0.109	0.126	-0.078	0.030	0.073	0.361
	*GC*	0.013	0.012	-0.007	0.004	0.058	0.000	13.897	0.001	0.092	0.053	0.023	0.022	0.036	0.137	0.039	-0.037	0.013	0.027	0.172
**ET**	*RC*	0.025	0.041	-0.015	0.000	0.132	0.024	0.189	0.045	0.622	0.696	0.354	0.259	0.035	0.375	0.087	-0.099	-0.026	0.009	0.697
	*GC*	0.006	0.014	-0.004	0.000	0.044	0.002	6.198	0.020	0.083	0.035	0.030	0.035	0.009	0.019	0.025	-0.021	-0.003	0.001	0.160
**GH**	*RC*	0.028	0.045	-0.029	0.016	0.136	0.011	0.484	-0.161	0.210	0.411	0.194	0.263	0.154	0.146	0.095	-0.129	-0.042	0.031	0.443
	*GC*	0.006	0.014	-0.007	0.009	0.047	0.001	12.372	-0.029	0.124	0.044	0.031	0.044	0.035	0.031	0.019	-0.021	-0.008	0.006	0.207
**GN**	*RC*	0.102	0.072	-0.076	-0.035	0.187	0.134	0.540	0.133	0.477	0.502	0.339	0.281	0.326	0.192	0.156	-0.103	0.042	0.074	0.597
	*GC*	0.021	0.020	-0.019	-0.019	0.047	0.006	12.917	0.019	0.096	0.020	0.038	0.055	0.068	0.059	0.017	-0.037	0.009	0.011	0.205
**KE**	*RC*	-0.129	0.082	0.037	-0.025	0.007	-0.087	0.249	-0.141	0.291	0.327	0.046	0.234	-0.060	0.075	-0.064	-0.129	0.014	0.077	0.668
	*GC*	-0.027	0.029	0.009	-0.013	0.003	-0.007	11.176	-0.077	0.112	0.067	0.006	0.022	-0.017	0.017	-0.010	-0.036	0.003	0.014	0.165
**LS**	*RC*	-0.021	-0.004	0.040	0.026	0.042	-0.065	0.320	-0.217	0.298	0.390	0.177	0.214	-0.022	0.025	0.000	-0.091	0.010	0.025	0.553
	*GC*	-0.005	-0.001	0.009	0.014	0.018	-0.006	11.755	-0.104	0.135	0.044	0.027	0.016	-0.010	0.012	0.000	-0.020	0.002	0.005	0.169
**LR**	*RC*	0.099	0.002	-0.016	-0.066	0.184	-0.003	0.406	-0.082	0.312	0.448	0.209	0.391	0.356	0.142	0.080	-0.056	0.019	0.035	0.535
	*GC*	0.015	0.001	-0.005	-0.029	0.050	0.000	11.098	-0.026	0.109	0.044	0.015	0.085	0.043	0.062	0.006	-0.017	0.006	0.007	0.194
**MW**	*RC*	0.040	-0.016	-0.008	-0.020	0.130	-0.123	0.446	-0.109	0.429	0.523	0.015	0.180	0.110	0.121	0.111	-0.068	0.019	-0.011	0.587
	*GC*	0.009	-0.006	-0.002	-0.010	0.037	-0.010	8.958	-0.070	0.108	0.032	0.003	0.026	0.022	0.019	0.015	-0.021	0.004	-0.002	0.119
**ML**	*RC*	0.051	0.046	-0.045	-0.008	0.092	0.203	0.506	0.072	0.528	0.509	0.287	0.226	0.070	0.285	0.043	-0.040	0.031	0.050	0.574
	*GC*	0.011	0.014	-0.011	-0.004	0.019	0.008	7.337	0.011	0.077	0.013	0.027	0.042	0.024	0.042	0.007	-0.013	0.005	0.006	0.198
**MZ**	*RC*	0.035	0.029	-0.052	-0.031	0.285	0.046	0.452	-0.043	0.591	0.597	0.386	0.240	-0.178	0.262	-0.015	-0.069	0.057	0.059	0.452
	*GC*	0.003	0.009	-0.014	-0.017	0.030	0.006	8.010	-0.026	0.077	0.043	0.041	0.029	-0.122	0.061	0.000	-0.024	0.015	0.011	0.126
**NE**	*RC*	0.086	0.019	-0.050	-0.037	0.265	0.117	0.611	0.223	0.676	0.421	0.046	0.086	0.017	0.463	0.237	-0.108	-0.047	0.029	0.713
	*GC*	0.016	0.006	-0.013	-0.021	0.051	0.004	7.313	0.030	0.065	0.028	0.005	0.015	0.006	0.018	0.037	-0.037	-0.007	0.003	0.159
**RW**	*RC*	0.014	0.020	-0.012	-0.035	0.079	-0.205	0.267	-0.058	0.436	0.641	0.172	0.493	0.096	0.172	0.045	-0.005	-0.039	-0.017	0.518
	*GC*	0.003	0.007	-0.002	-0.018	0.033	-0.014	5.427	-0.040	0.077	0.023	0.027	0.035	0.012	0.015	0.013	0.000	-0.004	-0.003	0.080
**ST**	*RC*	0.087	-0.029	-0.033	0.018	0.109	-0.282	0.392	-0.179	0.306	0.174	-0.116	-0.096	0.075	0.007	0.103	-0.095	-0.050	0.021	0.152
	*GC*	0.020	-0.009	-0.008	0.009	0.034	-0.029	9.758	-0.103	0.119	0.051	-0.014	-0.013	0.022	0.002	0.015	-0.020	-0.014	0.005	0.079
**SN**	*RC*	-0.035	0.048	0.006	-0.009	0.127	0.095	0.263	0.119	0.314	0.327	0.161	0.090	0.003	0.141	0.123	-0.227	-0.103	-0.007	0.370
	*GC*	-0.008	0.017	0.001	-0.005	0.053	0.003	13.810	0.029	0.084	0.045	0.024	0.017	0.001	0.018	0.037	-0.040	-0.011	-0.001	0.192
**SL**	*RC*	0.142	0.045	-0.067	-0.005	0.251	0.014	0.340	0.014	0.470	0.386	0.254	0.277	0.320	0.232	0.158	-0.091	0.066	0.089	0.537
	*GC*	0.022	0.015	-0.019	-0.002	0.061	0.001	9.636	0.002	0.129	0.039	0.013	0.050	0.061	0.065	0.014	-0.033	0.013	0.015	0.193
**SZ**	*RC*	-0.084	0.028	0.075	-0.010	-0.002	-0.120	0.252	-0.240	0.190	0.191	0.035	0.241	-0.101	0.016	-0.050	-0.117	-0.022	0.024	0.491
	*GC*	-0.025	0.010	0.015	-0.005	-0.001	-0.009	11.529	-0.082	0.109	0.040	0.005	0.018	-0.052	0.006	-0.012	-0.018	-0.005	0.005	0.135
**TZ**	*RC*	0.025	0.020	-0.019	0.005	0.111	-0.054	0.506	-0.007	0.563	0.616	0.422	0.362	0.176	0.001	0.093	-0.131	-0.028	0.034	0.611
	*GC*	0.006	0.007	-0.005	0.003	0.036	-0.005	10.678	-0.005	0.066	0.037	0.025	0.040	0.035	0.000	0.016	-0.033	-0.006	0.007	0.144
**UG**	*RC*	0.039	0.045	-0.022	0.004	0.152	-0.080	0.390	-0.122	0.369	0.252	-0.084	0.280	0.070	0.203	0.087	-0.062	-0.003	-0.002	0.665
	*GC*	0.008	0.014	-0.005	0.002	0.041	-0.009	12.008	-0.071	0.112	0.037	-0.027	0.028	0.017	0.051	0.012	-0.016	-0.001	0.000	0.132
**ZM**	*RC*	0.101	0.028	-0.066	0.000	0.190	0.003	0.447	-0.229	0.335	0.525	0.196	0.259	0.132	0.082	0.226	-0.119	-0.047	0.011	0.533
	*GC*	0.021	0.010	-0.017	0.000	0.060	0.000	17.060	-0.114	0.144	0.044	0.019	0.039	0.044	0.020	0.030	-0.037	-0.010	0.002	0.227
**ZW**	*RC*	0.000	0.027	-0.033	-0.014	0.112	-0.028	0.349	-0.304	0.128	0.535	0.120	0.204	-0.093	0.098	0.100	-0.196	-0.083	-0.007	0.568
	*GC*	0.000	0.010	-0.008	-0.007	0.037	-0.003	8.513	-0.083	0.091	0.032	0.022	0.028	-0.043	0.019	0.021	-0.021	-0.016	-0.001	0.171

The generalized concentration index for each independent variable was calculated as: $GC_{k}=\bar{X_{k}}\times RC_{k}$.

Based on the regression coefficients and generalized concentration index of each explanatory variable, we measured the contribution of each factor to the overall RC and GC as *β*_*k*_ × *GC*_*k*_/(*μ* - *μ*^2^) and *β*_*k*_ × *GC*_*k*_/1 - *μ*, respectively. The “contribution” indicates how much of the association between wealth and HIV/AIDS in each country is explained by variation in a given explanatory factor among different socioeconomic groups. A positive contribution of a given factor to the RC and GC suggests that the socioeconomic distribution of the factor and the association of the relevant factor with HIV/AIDS contribute to a greater prevalence of HIV/AIDS among wealthier respondents and *vice versa*.

**Figure 4:**
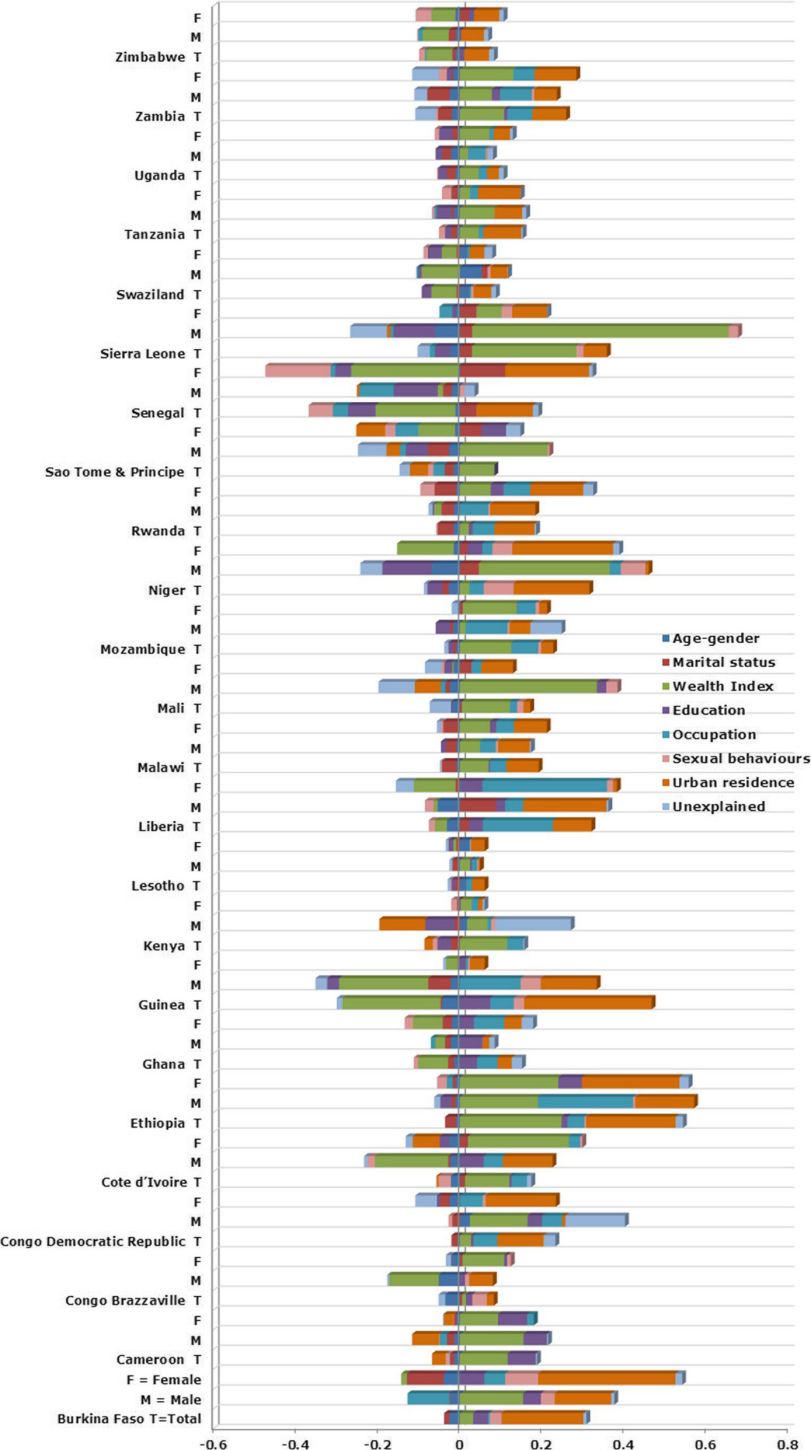
Contribution of each factor to the relative inequality of HIV/AIDS prevalence in SSA region.

**Figure 5:**
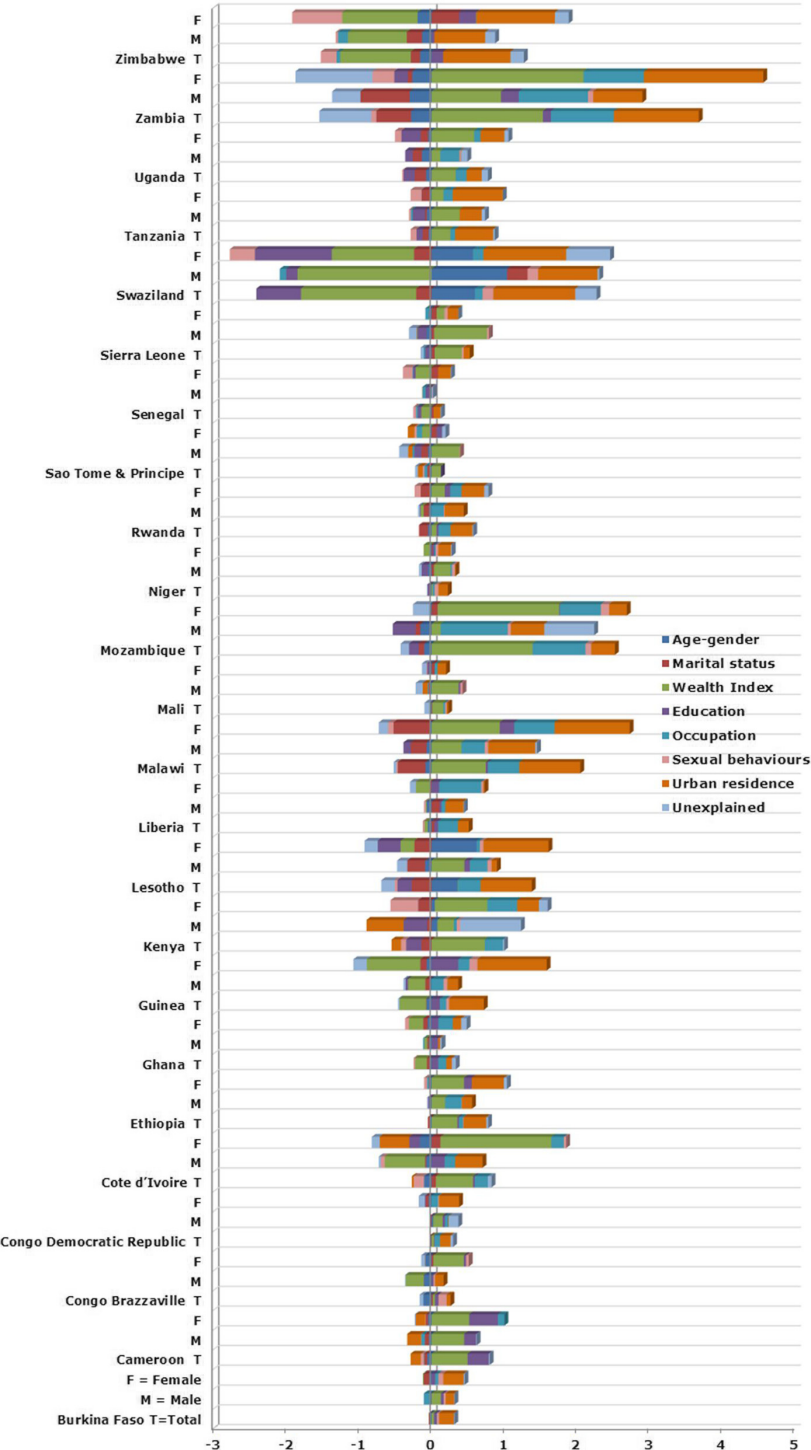
Contribution of each factor to the absolute inequality of HIV/AIDS prevalence in SSA region.

Figure 4 and 5 illustrate the overall contribution of each category to the relative and generalized socioeconomic inequality in HIV/AIDS prevalence for the total population, as well as for men and women separately (for detailed contribution of each factor see Additional file 1). Wealth contributed positively to HIV/AIDS, independently of other determinants of socioeconomic inequality, in the majority of the SSA countries. However, wealth contributed negatively to the relative inequality in Senegal and Guinea (see Figure 4), and to the absolute inequality in Swaziland and Zimbabwe (see Figure 5). Based on the decomposition results for the total population, wealth made a significant percentage contribution (calculated as its contribution multiplied by 100 and divided by the RC or GC) to socioeconomic inequalities in HIV/AIDS prevalence, either measured in absolute or relative terms, across SSA countries (median = 49%, interquartile range [IQR] = 90%).

Apart from wealth, urban residence increased the absolute and relative concentration of HIV/AIDS among wealthier individuals in the majority of countries. According to the decomposition results of the RC and GC for the total population, the median percentage contribution of urban residence to wealth-based inequality in HIV/AIDS across the 24 countries was 54% (IQR = 81%). In general, occupation status also contributed to the concentration of HIV/AIDS among the better-off. The education factor increased the concentration of HIV/AIDS prevalence among poorer individuals in Swaziland and Lesotho. This factor, however, increased the concentration of HIV/AIDS among the rich in Cameroon. Sexual behaviours, in general, did not contribute significantly to observed SES inequalities in HIV/AIDS in SSA countries.

## Discussion and conclusions

We used data from the DHS and AIDS Indicator Surveys to measure inequalities in HIV/AIDS prevalence in 24 SSA countries. The generalized concentration index was used to quantify and decompose wealth-based inequalities in HIV/AIDS for the whole population, for men and women, as well as for urban and rural regions in each country. Our results suggested that HIV/AIDS is more prevalent among relatively wealthier countries and individuals in the SSA region. Separate analysis by gender also showed HIV/AIDS was concentrated among wealthier men and women in the majority of countries. These results confirm the findings of recent studies [5,18,24,55,56] showing higher concentration of HIV/AIDS prevalence among socioeconomically advantaged individuals in some SSA countries. Our findings for urban areas showed that the HIV/AIDS was more prevalent among the poor in countries such as Uganda, Kenya, Zimbabwe and Swaziland. However, in rural areas, HIV/AIDS was concentrated among wealthier individuals in the majority of countries. These findings support a recent study by Magadi [57] suggesting that poorer individuals in urban areas in SSA face comparative disadvantage with respect of HIV/AIDS prevalence. These results, thus, suggest that the positive association between wealth and HIV/AIDS that we found for whole population in the majority of SSA countries reflects the situation in rural regions where most people reside.

Results from our decomposition analyses showed that, aside from wealth *per se*, urban residence was the most important factor contributing to the relative and absolute concentration of HIV/AIDS prevalence among the better-off. Urban residents were wealthier than their rural counterparts (as indicated by the positive values of the *RC*_*k*_ and *GC*_*k*_ in Table 6). Additionally, living in urban areas was positively associated with the probability of being HIV positive (as indicated by the positive value of the coefficient, *β*, in Table 5). Further work is needed to clarify the mechanisms through which urban residence influences the prevalence of HIV/AIDS. One possibility is that the positive association between urban residence and prevalence of HIV/AIDS is not explained by increased incidence, but rather by improved access to treatment (and survival conditional on infection) among urban compared to rural populations. For example, a study by Zungu-Dirwayi and colleagues [58] that examined HIV/AIDS prevention programs in six SSA countries (Botswana, Lesotho, Mozambique, South Africa, Swaziland and Zimbabwe) showed that the provision of services such as voluntary counseling and testing were lower in rural compared with urban areas.

Our findings indicated that wealth-based inequalities in HIV/AIDS persisted after accounting for other demographic characteristics, region of residence, number of partners and early sexual activity. Household wealth was associated with higher prevalence of HIV/AIDS in most SSA countries. Unfortunately, it was not possible to examine differences in the frequency or quality of HIV treatment, or details on HIV prevention. The concentration of HIV/AIDS among wealthier men and women may be explained by behavioural differences [56]. Our results indicated that wealthier individuals (both men and women) in SSA countries reported more sexual partners than their poorer counterparts (see the positive value of the *RC*_*k*_ and *GC*_*k*_ for the number of sexual partners in Table 6). The concentration of HIV/AIDS among wealthier individuals may also be partially explained by unsafe sexual practices. Another potential explanation for the higher prevalence of HIV/AIDS among wealthier individuals is that socially advantaged individuals are more likely to receive treatment for HIV/AIDS, extending their survival relative to poorer individuals [25,56].

The direction of the association between wealth and HIV/AIDS was not consistent across all sampled countries. For example, there was a negative association between wealth and HIV/AIDS in Swaziland and Zimbabwe. The higher absolute concentration of HIV/AIDS among poorer individuals in Swaziland may be explained by cultural beliefs that discourage safe sexual practices, including monogamous relationships and condom use [59], and the concentration of these behaviours among socioeconomically disadvantaged groups. Based on the DHS 2006/07, for example, around 17 per cent of individuals who had sex with someone other than their spouse in Swaziland did not use a condom and this behaviour was more common among poorer individuals (GC = -2.056, CI: -1.60 -2.52). Recent studies by Asiedu and colleagues [23] and Fox [17] have also demonstrated that poorer individuals in Swaziland are at higher risk of being HIV positive than their wealthier counterparts. Similarly, based on the DHS 2010/11, socioeconomically disadvantaged groups in Zimbabwe reported a higher prevalence of unsafe sexual practices with sexual partners outside their marriage compared to wealthier individuals (GC = -0.348, CI: -0.30 -0.39).

There were limitations to our study. First, although we used the most recent available survey data set for each country to investigate socioeconomic inequality in HIV/AIDS, the DHSs were conducted in different years and inequality estimates might have changed with the time of survey. Second, although self-reported sexual behaviours are likely measured with error [23,60], we included these variables in the decomposition analysis because these sexual practices are associated with HIV infection (e.g., [6,26]) and may also influence levels of household wealth [61,62]. Sensitivity analyses excluding these variables yielded very similar results. Third, our analyses were based on cross-sectional data and it was not possible to establish temporality between explanatory factors and HIV status, limiting causal inference. For example, marital status might influence the probability of HIV infection; in turn, having HIV/AIDS might influence marital status. Thus, our results can be interpreted in terms of observed associations between explanatory variables and HIV/AIDS status. Fourth, the unexplained component in the decomposition analysis contributed significantly to socioeconomic inequalities in HIV/AIDS in some countries although this was not the case in most. This suggests that unmeasured explanatory factors other than those included in the model influence socioeconomic inequalities in HIV/AIDS. Fifth, women may have limited financial autonomy within a household and therefore wealth may be an imperfect proxy for individual SES. Finally, as the variable of interest in this study is binary, the minimum and maximum of the GC are not – *μ* and *μ* and depend on the mean of the variable [46]. There is lively debate in the health economics literature as to whether Wagstaff’s correction or Erreygers’ Index, which suggests multiplying the concentration index by 4 *μ*[63-66], is a better method for correcting the GC when the outcome variable is bounded. Nevertheless, our sensitivity analyses using Erreygers’ correction in the estimations of the GC yielded qualitatively similar inference.

In conclusion, our findings demonstrate substantial variation among SSA countries in the magnitude of relative and absolute socioeconomic inequalities in HIV/AIDS. Unlike the distribution of other health outcomes, HIV/AIDS was generally concentrated among wealthier countries and individuals. This may be due to greater incidence of disease but more effective treatment and thus better outcomes could also be contributing. Our results also suggested a statistically significant negative association between the RC/SRC for HIV/AIDS prevalence and GDP per capita and Gini index across countries. This suggests that HIV/AIDS is relatively less concentrated among wealthier individuals in countries with higher living standards and greater income inequality. Further, we found a positive association between the GC and Southern Africa countries, suggesting that absolute inequality for HIV/AIDS is greater in these countries. Beside wealth, other important contributors to socioeconomic inequalities included urban residence and occupation status. Results also indicated substantial variation in the factors explaining socioeconomic inequalities in HIV/AIDS prevalence across SSA countries. In future work, particular attention should be devoted to understanding the mechanisms by which HIV/AIDS is concentrated among wealthier individuals and urban residents. It is crucial to understand to what extent this is indicative of the success of better treatment which needs to be spread to the entire population and to what extent it is caused by inadequate prevention.

## Competing interests

The authors declare that they have no competing interests.

## Authors’ contribution

All authors contributed to the conception and design of the study, MH performed the statistical analysis, all authors interpreted results, MH drafted the manuscript, and DS, JH and AN helped with drafting and revisions. All authors read and approved the final version of the manuscript.

## Supplementary Material

Additional file 1: Appendix 1 Contribution of each factor to the relative inequality of HIV/AIDS prevalence in SSA region (detailed results). **Appendix 2.** Contribution of each factor to the absolute inequality of HIV/AIDS prevalence in SSA region (detailed results). Click here for file

## References

[B1] Marmot M Social determinants of health inequalities Lancet 2005 365 1099 1104 10.1016/S0140-6736(05)71146-6 15781105

[B2] Braveman P Tarimo E Social inequalities in health within countries: not only an issue for affluent nations Soc Sci Med 2002 54 1621 1635 10.1016/S0277-9536(01)00331-8 12113445

[B3] CSDH Closing the Gap in a Generation: Health Equity through Action on the Social Determinants of Health 2008 Geneva: Final Report of the Commission on Social Determinants of Health, World Health Organization 10.1016/S0140-6736(08)61690-6 18994664

[B4] World Health Organization Global Report: UNAIDS Report on the Global AIDS Epidemic 2010 2010 Geneva: WHO

[B5] Fortson JG The gradient in sub-Saharan Africa: socioeconomic status and HIV/AIDS Demography 2008 45 303 322 10.1353/dem.0.0006 18613483 PMC2831364

[B6] Magadi MA Understanding the gender disparity in HIV infection across countries in sub-Saharan Africa: evidence from the Demographic and Health Surveys Soc Heal Illn 2011 33 522 539 10.1111/j.1467-9566.2010.01304.x PMC3412216 21545443

[B7] Masanjala W The poverty-HIV/AIDS nexus in Africa: a livelihood approach Soc Sci Med 2007 64 1032 1041 10.1016/j.socscimed.2006.10.009 17126972

[B8] Gillespie S Kadiyala S Greener R Is poverty or wealth driving HIV transmission? AIDS 2007 21 Suppl 7 S5 S16 10.1097/01.aids.0000300531.74730.72 18040165

[B9] Andoh SY Umezaki M Nakamura K Kizuki M Takano T Correlation between national income, HIV/AIDS and political status and mortalities in African countries Publ Health 2006 120 624 633 10.1016/j.puhe.2006.04.008 16753194

[B10] Kongnyuy EJ Wiysonge CS Mbu RE Nana P Kouam L Wealth and sexual behaviour among men in Cameroon BMC Int Health Hum Right 2006 6 11 10.1186/1472-698X-6-11 PMC1574345 16965633

[B11] Awusabo-Asare K Annim SK Wealth status and risky sexual behaviour in Ghana and Kenya Appl Health Econ Health Pol 2008 6 27 39 10.2165/00148365-200806010-00003 18774868

[B12] Wang W Sulzbach S De S Utilization of HIV-related services from the private health sector: a multi-country analysis Soc Sci Med 2011 72 216 223 10.1016/j.socscimed.2010.11.011 21145151

[B13] De Walque D How does the impact of an HIV/AIDS information campaign vary with educational attainment? Evidence from rural Uganda J Dev Econ 2007 84 686 714 10.1016/j.jdeveco.2006.12.003

[B14] Glynn JR Caraël M Buvé A Anagonou S Zekeng L Kahindo M Musonda R Does increased general schooling protect against HIV infection? A study in four African cities Trop Med Int Heal 2004 9 4 14 10.1046/j.1365-3156.2003.01168.x 14728602

[B15] Hargreaves JR Glynn JR Educational attainment and HIV-1 infection in developing countries: a systematic review Trop Med Int Heal 2002 7 489 498 10.1046/j.1365-3156.2002.00889.x 12031070

[B16] Fox AM The social determinants of HIV serostatus in sub-Saharan Africa: an inverse relationship between poverty and HIV? Public Health Rep 2010 125 Suppl 4 16 24 20629252 10.1177/00333549101250S405 PMC2882971

[B17] Fox AM The HIV poverty thesis re-examined: Poverty, wealth or inequality as a social determinant of HIV in sub-Saharan Africa? J Biosoc Sci 2012 44 459 480 10.1017/S0021932011000745 22273351

[B18] Mishra V Assche SB-V Greener R Vaessen M Hong R Ghys PD Boerma JT Van Assche A Khan S Rutstein SO HIV infection does not disproportionately affect the poorer in sub-Saharan Africa AIDS 2007 21 Suppl 7 S17 S28 10.1097/01.aids.0000300532.51860.2a 18040161

[B19] UNAIDS UNAIDS Report on the Global AIDS Epidemic 2012 Volume 2008 2012 Geneva: Joint United Nations Programme on HIV/AIDS: World Health Organization 106 Report

[B20] Over M Piot P Jamison DT, Mosley WH, Measham AR, Bobadilla JL HIV Infection and Sexually Transmitted Diseases Dis Control Priorities Dev Ctries 1993 New York: Oxford University Press 455 527

[B21] Gregson S Waddell H Chandiwana S School education and HIV control in sub-Saharan Africa: from discord to harmony? J Int Dev 2001 13 467 485 10.1002/jid.798

[B22] Iorio D Santaeulalia-Llopis R Education, HIV status, and risky sexual behavior: how much does the stage of the HIV epidemic matter? 2012 Working Paper 624, Barcelona Graduate School of Economics (GSE)

[B23] Asiedu C Asiedu E Owusu F The socio-economic determinants of HIV/AIDS infection rates in Lesotho, Malawi, Swaziland and Zimbabwe Dev Pol Rev 2012 30 305 326 10.1111/j.1467-7679.2012.00578.x

[B24] Shelton JD Cassell MM Adetunji J Is poverty or wealth at the root of HIV? Lancet 2005 366 1057 1058 10.1016/S0140-6736(05)67401-6 16182881

[B25] De Walque D Who Gets AIDS and How? The Determinants of HIV Infection and Sexual Behaviors in Burkina Faso, Cameroon, Ghana, Kenya, and Tanzania 2006 Washington, DC: World Bank Publications

[B26] Magadi MA Desta M A multilevel analysis of the determinants and cross-national variations of HIV seropositivity in sub-Saharan Africa: evidence from the DHS Heal Place 2011 17 1067 1083 10.1016/j.healthplace.2011.06.004 PMC3248638 21741295

[B27] Tang CS Wong CY Lee AM Gender-related psychosocial and cultural factors associated with condom use among Chinese married women AIDS Educ Prev 2001 13 329 342 10.1521/aeap.13.4.329.21426 11565592

[B28] Berhan A Berhan Y A meta-analysis on higher-risk sexual behavior of women in 28 third world countries World J AIDS 2012 2 78 88 10.4236/wja.2012.22011

[B29] Wamoyi J Wight D Plummer M Mshana GH Ross D Transactional sex amongst young people in rural northern Tanzania: an ethnography of young women’s motivations and negotiation Reprod Health 2010 7 2 10.1186/1742-4755-7-2 20429913 PMC2867784

[B30] Burke M Gong E Jones K Income shocks and HIV in Sub-Saharan Africa IFPRI - Discussion Paper 1146 2011 International Food Policy Research Institute

[B31] Wagstaff A Paci P Van Doorslaer E On the measurement of inequalities in health Soc Sci Med 1991 33 545 557 10.1016/0277-9536(91)90212-U 1962226

[B32] Corsi DJ Neuman M Finlay JE Subramanian SV Demographic and health surveys: a profile Int J Epidemiol 2012 41 1602 1613 10.1093/ije/dys184 23148108

[B33] Rutstein SO Rojas G Guide to DHS Statistics Heal San Fr 2006 171

[B34] Demographic and Health Survey Demographic and Health Survey Interviewer’s Manual 2006 ORC Macro: Calverton, MD

[B35] Subramanian SV Perkins JM Özaltin E George DS Weight of nations: a socioeconomic analysis of women in low- to middle-income countries Am J Clin Nutr 2011 93 413 421 10.3945/ajcn.110.004820 21068343 PMC3021433

[B36] World Bank World Development Indicators database and Global Development Finance http://databank.worldbank.org/data/home.aspx

[B37] World Bank Worldwide Governance Indicators http://data.worldbank.org/data-catalog/worldwide-governance-indicators

[B38] Filmer D Pritchett LH Estimating wealth effects without expenditure data–or tears: an application to educational enrollments in states of India Demography 2001 38 115 132 11227840 10.1353/dem.2001.0003

[B39] Rutstein SO Johnson K The DHS Wealth Index. DHS Comparative Reports No. 6 2004 Calverton: ORC Macro, MEASURE DHS

[B40] Mazumdar S Determinants of inequality in child malnutrition in India: the poverty-undernutrition linkage Asian Popul Stud 2010 6 307 333 10.1080/17441730.2010.512763

[B41] Mayer-Foulkes D Larrea C Racial and Ethnic Health Inequities: Bolivia 2005 Guatemala and Peru: Brazil

[B42] Kakwani N Wagstaff A van Doorslaer E Socioeconomic inequalities in health: measurement, computation, and statistical inference J Econom 1997 77 87 103 10.1016/S0304-4076(96)01807-6

[B43] O’Donnell O van Doorslaer E Wagstaff A Lindelow M Analyzing Health Equity Using Household Survey Data - A Guide to Techniques and Their Implementation 2008 Geneva: The World Bank

[B44] Newey WK West KD Automatic lag selection in covariance matrix estimation Rev Econ Stud 1994 61 631 653 10.2307/2297912

[B45] World Bank Quantitative Techniques for Health Equity Analysis: The Concentration Index 2012 Technical Note Number 7, http://siteresources.worldbank.org/INTPAH/Resources/Publications/Quantitative-Techniques/health_eq_tn07.pdf

[B46] Wagstaff A The bounds of the concentration index when the variable of interest is binary, with an application to immunization inequality Health Econ 2005 14 429 432 10.1002/hec.953 15495147

[B47] Mackenbach JP Kunst AE Cavelaars AEJM Groenhof F Geurts JJM Andersen O Bonte JTP Borgan JK Crialesi R Desplanques G Filakti H Harding S Grotvedt L Helmert U Junker C Lahelma E Lundberg O Martikainen P Matheson J Mielck A Minder CE Mizrahi A Pagnanelli F Rasmussen N Regidor E Spuhler T Valkonen T Socioeconomic inequalities in morbidity and mortality in western Europe Lancet 1997 349 1655 1659 10.1016/S0140-6736(96)07226-1 9186383

[B48] Ásgeirsdóttir TL Ragnarsdóttir DÓ Determinants of relative and absolute concentration indices: evidence from 26 European countries Int J Equity Health 2013 12 53 10.1186/1475-9276-12-53 23866925 PMC3726484

[B49] Altman DG Bland JM Interaction revisited: the difference between two estimates BMJ 2003 326 219 10.1136/bmj.326.7382.219 12543843 PMC1125071

[B50] Tsafack Temah C The role of income and gender inequalities in the spread of The HIV/AIDS epidemic: evidence from Sub-Saharan Africa 2008 Clermont-Ferrand, France: Doctoral dissertation, University d’Auvergne

[B51] Menon-Johansson AS Good governance and good health: the role of societal structures in the human immunodeficiency virus pandemic BMC Int Health Hum Right 2005 5 4 10.1186/1472-698X-5-4 PMC1112596 15850480

[B52] Wagstaff A van DE Watanabe N On decomposing the causes of health sector inequalities with an application to malnutrition inequalities in Vietnam J Econom 2003 112 207 10.1016/S0304-4076(02)00161-6

[B53] O’Donnell O Van Doorslaer E Wagstaff A Decomposition of inequalities in health and health care Elgar Companion to Heal Econ 2006 Cheltenham: Edward Elgar Publishing 179 192

[B54] Van De Poel E O’Donnell O Van Doorslaer E Urbanization and the spread of diseases of affluence in China Econ Hum Biol 2009 7 200 216 10.1016/j.ehb.2009.05.004 19560989

[B55] Lachaud J-P HIV prevalence and poverty in Africa: micro- and macro-econometric evidences applied to Burkina Faso J Health Econ 2007 26 483 504 10.1016/j.jhealeco.2006.10.007 17113173

[B56] Msisha WM Kapiga SH Earls F Subramanian SV Socioeconomic status and HIV seroprevalence in Tanzania: a counterintuitive relationship Int J Epidemiol 2008 37 1297 1303 10.1093/ije/dyn186 18820319 PMC2638871

[B57] Magadi MA The disproportionate high risk of HIV infection among the urban poor in sub-Saharan Africa AIDS Behav 2013 17 1645 1654 10.1007/s10461-012-0217-y 22660933 PMC3663197

[B58] Zungu-Dirwayi N Shisana O Udjo E Mosala T Seager J An Audit of HIV/Aids Policies: In Botswana, Lesotho, Mozambique, South Africa, Swaziland, And Zimbabwe 2004 Cape Town: Human Sciences Research Council

[B59] Integrated Regional Information Networks Swaziland: A culture that encourages HIV/AIDS http://www.refworld.org/docid/49e6ef2dc.html

[B60] Curtis SL Sutherland EG Measuring sexual behaviour in the era of HIV/AIDS: the experience of Demographic and Health Surveys and similar enquiries. [Miscellaneous] In, Sex Transm Infect 2004 80 ii22 ii27 10.1136/sti.2004.011650 PMC1765855 15572636

[B61] Leclerc-Madlala S Youth, HIV/AIDS and the importance of sexual culture and context Soc Dyn 2002 28 20 41 10.1080/02533950208458721

[B62] Leclerc-Madlala S Transactional sex and the pursuit of modernity Soc Dyn 2003 29 213 233 10.1080/02533950308628681

[B63] Erreygers G Correcting the concentration index J Health Econ 2009 28 504 515 10.1016/j.jhealeco.2008.02.003 18367273

[B64] Wagstaff A Reply to guido erreygers and Tom Van Ourti’s comment on “The concentration index of a binary outcome revisited” Health Econ 2011 20 1166 1168 10.1002/hec.1753 21674678

[B65] Erreygers G Van Ourti T Measuring socioeconomic inequality in health, health care and health financing by means of rank-dependent indices: a recipe for good practice J Health Econ 2011 30 685 694 10.1016/j.jhealeco.2011.04.004 21683462 PMC3158909

[B66] Wagstaff A Correcting the concentration index: a comment J Health Econ 2009 28 516 520 author reply 521–524 10.1016/j.jhealeco.2008.12.003 19167117

