# Supplementary material for: Socioeconomic inequalities in HIV/AIDS prevalence in sub-Saharan African countries: evidence from the Demographic Health Surveys
Source: Int J Equity Health. 2014 Feb 18;13:18. doi: 10.1186/1475-9276-13-18 (PMC3930550; doi:10.1186/1475-9276-13-18)
Supplement: Additional file 1: Appendix 1 — Contribution of each factor to the relative inequality of HIV/AIDS prevalence in SSA region (detailed results). Appendix 2. Contribution of each factor to the absolute inequality of HIV/AIDS prevalence in SSA region (detailed results). [file 1475-9276-13-18-S1.docx]

**Appendix 1.** Contribution of each factor to the relative inequality of HIV/AIDS prevalence in SSA region (detailed results)

| Country |  | Age-gender | Marital status | Wealth Index | Education | Occupation | Sexual Behaviours | Urban Residence | Unexplained | RC |
| --- | --- | --- | --- | --- | --- | --- | --- | --- | --- | --- |
| BF | Total | -0.026 | -0.013 | 0.032 | 0.036 | 0.004 | 0.029 | 0.199 | 0.007 | 0.269 |
|  | Male | -0.023 | -0.002 | 0.154 | 0.044 | -0.102 | 0.033 | 0.138 | 0.008 | 0.248 |
|  | Female | -0.039 | -0.089 | -0.014 | 0.059 | 0.051 | 0.080 | 0.334 | 0.016 | 0.398 |
| CM | Total | -0.014 | -0.010 | 0.116 | 0.068 | -0.001 | -0.010 | -0.033 | 0.003 | 0.120 |
|  | Male | -0.013 | -0.018 | 0.155 | 0.057 | -0.017 | -0.001 | -0.066 | 0.003 | 0.098 |
|  | Female | -0.009 | -0.006 | 0.093 | 0.071 | 0.016 | -0.001 | -0.023 | -0.003 | 0.138 |
| CG | Total | -0.036 | 0.005 | 0.010 | 0.015 | - | 0.036 | 0.017 | -0.016 | 0.030 |
|  | Male | -0.051 | -0.001 | -0.120 | 0.012 | - | 0.010 | 0.058 | -0.005 | -0.097 |
|  | Female | -0.021 | 0.006 | 0.102 | 0.007 | - | 0.009 | -0.001 | -0.013 | 0.090 |
| CD | Total | -0.003 | -0.018 | 0.027 | 0.006 | 0.058 | 0.000 | 0.113 | 0.029 | 0.211 |
|  | Male | 0.024 | -0.018 | 0.140 | 0.035 | 0.049 | -0.010 | 0.008 | 0.145 | 0.374 |
|  | Female | -0.025 | -0.023 | 0.000 | -0.007 | 0.055 | 0.007 | 0.171 | -0.053 | 0.125 |
| CI | Total | -0.022 | 0.012 | 0.108 | 0.005 | 0.038 | -0.030 | -0.005 | 0.011 | 0.117 |
|  | Male | -0.023 | -0.005 | -0.179 | 0.058 | 0.047 | -0.017 | 0.121 | -0.009 | -0.008 |
|  | Female | -0.026 | 0.019 | 0.245 | -0.024 | 0.028 | 0.005 | -0.065 | -0.018 | 0.166 |
| ET | Total | -0.009 | -0.027 | 0.247 | 0.014 | 0.041 | 0.004 | 0.218 | 0.018 | 0.507 |
|  | Male | -0.011 | -0.010 | 0.189 | -0.027 | 0.232 | 0.005 | 0.144 | -0.015 | 0.507 |
|  | Female | -0.010 | -0.008 | 0.239 | 0.057 | -0.014 | -0.024 | 0.238 | 0.023 | 0.501 |
| GH | Total | -0.014 | -0.015 | -0.073 | 0.041 | 0.050 | -0.010 | 0.035 | 0.025 | 0.038 |
|  | Male | -0.022 | -0.014 | -0.024 | 0.055 | -0.012 | 0.001 | 0.016 | 0.013 | 0.013 |
|  | Female | -0.019 | -0.023 | -0.073 | 0.034 | 0.074 | -0.020 | 0.043 | 0.028 | 0.044 |
| GN | Total | -0.044 | -0.003 | -0.239 | 0.073 | 0.058 | 0.025 | 0.311 | -0.014 | 0.167 |
|  | Male | -0.022 | -0.055 | -0.217 | -0.029 | 0.147 | 0.049 | 0.137 | -0.029 | -0.019 |
|  | Female | -0.003 | -0.003 | -0.028 | 0.014 | 0.006 | 0.004 | 0.036 | -0.007 | 0.019 |
| KE | Total | 0.000 | -0.022 | 0.115 | -0.033 | 0.039 | -0.011 | -0.020 | 0.003 | 0.070 |
|  | Male | 0.017 | -0.013 | 0.049 | -0.071 | 0.009 | 0.010 | -0.112 | 0.184 | 0.073 |
|  | Female | 0.002 | -0.007 | 0.027 | 0.000 | 0.015 | -0.014 | 0.011 | 0.005 | 0.039 |
| LS | Total | 0.016 | -0.012 | 0.000 | -0.009 | 0.013 | -0.002 | 0.031 | -0.008 | 0.030 |
|  | Male | -0.005 | -0.013 | 0.024 | 0.004 | 0.013 | 0.003 | 0.004 | -0.008 | 0.023 |
|  | Female | 0.023 | -0.009 | -0.007 | -0.012 | 0.002 | 0.002 | 0.033 | -0.007 | 0.026 |
| LR | Total | -0.031 | 0.022 | -0.029 | 0.033 | 0.171 | -0.014 | 0.094 | -0.001 | 0.244 |
|  | Male | -0.054 | 0.087 | -0.009 | 0.022 | 0.044 | -0.022 | 0.203 | 0.005 | 0.276 |
|  | Female | 0.004 | -0.011 | -0.102 | 0.050 | 0.304 | 0.015 | 0.010 | -0.043 | 0.226 |
| MW | Total | -0.008 | -0.036 | 0.070 | 0.003 | 0.040 | -0.002 | 0.079 | -0.004 | 0.143 |
|  | Male | -0.008 | -0.026 | 0.049 | -0.012 | 0.038 | 0.006 | 0.077 | 0.003 | 0.127 |
|  | Female | -0.001 | -0.039 | 0.073 | 0.015 | 0.043 | -0.006 | 0.080 | -0.010 | 0.155 |
| ML | Total | -0.019 | 0.005 | 0.117 | -0.003 | 0.018 | 0.016 | 0.017 | -0.052 | 0.097 |
|  | Male | -0.025 | -0.010 | 0.333 | 0.023 | -0.012 | 0.027 | -0.063 | -0.088 | 0.185 |
|  | Female | -0.015 | 0.028 | -0.003 | -0.020 | 0.024 | -0.006 | 0.077 | -0.041 | 0.044 |
| MZ | Total | -0.009 | -0.007 | 0.125 | -0.012 | 0.066 | 0.007 | 0.029 | -0.011 | 0.188 |
|  | Male | -0.017 | -0.007 | 0.014 | -0.035 | 0.102 | 0.005 | 0.051 | 0.076 | 0.188 |
|  | Female | -0.002 | 0.007 | 0.132 | 0.001 | 0.045 | 0.009 | 0.019 | -0.018 | 0.192 |
| NE | Total | -0.028 | -0.016 | 0.023 | -0.036 | 0.035 | 0.073 | 0.185 | -0.009 | 0.228 |
|  | Male | -0.069 | 0.046 | 0.319 | -0.120 | 0.028 | 0.060 | 0.008 | -0.054 | 0.217 |
|  | Female | -0.015 | 0.018 | -0.138 | 0.036 | 0.025 | 0.048 | 0.246 | 0.015 | 0.235 |
| RW | Total | -0.016 | -0.038 | 0.021 | 0.009 | 0.053 | -0.004 | 0.098 | 0.004 | 0.128 |
|  | Male | -0.015 | -0.030 | -0.018 | -0.003 | 0.069 | 0.004 | 0.111 | -0.011 | 0.107 |
|  | Female | -0.009 | -0.052 | 0.075 | 0.031 | 0.065 | -0.035 | 0.129 | 0.024 | 0.228 |
| ST | Total | -0.015 | -0.022 | 0.084 | 0.000 | -0.028 | -0.013 | -0.044 | -0.025 | -0.063 |
|  | Male | -0.026 | -0.052 | 0.214 | -0.053 | -0.014 | 0.005 | -0.033 | -0.070 | -0.030 |
|  | Female | -0.012 | 0.052 | -0.090 | 0.060 | -0.056 | -0.025 | -0.070 | 0.035 | -0.105 |
| SN | Total | -0.011 | 0.040 | -0.195 | -0.067 | -0.037 | -0.059 | 0.138 | 0.013 | -0.177 |
|  | Male | -0.020 | -0.021 | -0.013 | -0.108 | -0.084 | 0.011 | -0.006 | 0.024 | -0.215 |
|  | Female | -0.003 | 0.110 | -0.262 | -0.039 | -0.012 | -0.159 | 0.204 | 0.009 | -0.151 |
| SL | Total | -0.023 | 0.029 | 0.254 | -0.038 | -0.012 | 0.017 | 0.057 | -0.030 | 0.255 |
|  | Male | -0.060 | 0.030 | 0.625 | -0.101 | -0.007 | 0.022 | -0.008 | -0.090 | 0.410 |
|  | Female | -0.010 | 0.040 | 0.062 | -0.009 | -0.031 | 0.025 | 0.085 | 0.001 | 0.163 |
| SZ | Total | 0.023 | -0.008 | -0.061 | -0.024 | 0.004 | 0.006 | 0.044 | 0.011 | -0.005 |
|  | Male | 0.053 | 0.014 | -0.094 | -0.008 | -0.005 | 0.007 | 0.041 | 0.001 | 0.011 |
|  | Female | 0.018 | -0.008 | -0.036 | -0.034 | 0.005 | -0.011 | 0.037 | 0.019 | -0.010 |
| TZ | Total | -0.007 | -0.014 | 0.045 | -0.015 | 0.012 | -0.015 | 0.092 | 0.004 | 0.102 |
|  | Male | -0.014 | -0.005 | 0.084 | -0.037 | -0.006 | -0.006 | 0.067 | 0.010 | 0.093 |
|  | Female | -0.004 | -0.016 | 0.024 | -0.001 | 0.019 | -0.023 | 0.104 | 0.000 | 0.104 |
| UG | Total | -0.010 | -0.021 | 0.045 | -0.021 | 0.021 | -0.003 | 0.029 | 0.012 | 0.051 |
|  | Male | -0.022 | -0.021 | 0.019 | -0.017 | 0.043 | 0.003 | 0.001 | 0.014 | 0.021 |
|  | Female | -0.006 | -0.013 | 0.071 | -0.032 | 0.010 | -0.011 | 0.040 | 0.007 | 0.067 |
| ZM | Total | -0.020 | -0.034 | 0.108 | 0.008 | 0.061 | -0.005 | 0.082 | -0.050 | 0.150 |
|  | Male | -0.024 | -0.055 | 0.078 | 0.020 | 0.077 | 0.006 | 0.055 | -0.032 | 0.125 |
|  | Female | -0.016 | -0.004 | 0.130 | -0.011 | 0.052 | -0.019 | 0.102 | -0.066 | 0.167 |
| ZW | Total | -0.010 | -0.008 | -0.063 | 0.010 | -0.003 | -0.014 | 0.061 | 0.012 | -0.017 |
|  | Male | -0.010 | -0.017 | -0.064 | 0.003 | -0.011 | -0.003 | 0.055 | 0.011 | -0.035 |
|  | Female | -0.011 | 0.021 | -0.059 | 0.013 | 0.000 | -0.039 | 0.061 | 0.011 | -0.002 |

**Appendix 2.** Contribution of each factor to the absolute inequality of HIV/AIDS prevalence in SSA region (detailed results)

| Country |  | Age-gender | Marital status | Wealth Index | Education | Occupation | Sexual Behaviours | Urban Residence | Unexplained | GC |
| --- | --- | --- | --- | --- | --- | --- | --- | --- | --- | --- |
| BF | Total | -0.026 | -0.013 | 0.033 | 0.037 | 0.004 | 0.029 | 0.203 | 0.007 | 0.274 |
|  | Male | -0.019 | -0.002 | 0.129 | 0.036 | -0.086 | 0.028 | 0.116 | 0.006 | 0.208 |
|  | Female | -0.033 | -0.075 | -0.012 | 0.049 | 0.043 | 0.067 | 0.280 | 0.014 | 0.333 |
| CM | Total | -0.058 | -0.042 | 0.493 | 0.291 | -0.004 | -0.043 | -0.142 | 0.014 | 0.510 |
|  | Male | -0.038 | -0.053 | 0.446 | 0.165 | -0.050 | -0.004 | -0.191 | 0.008 | 0.283 |
|  | Female | -0.048 | -0.031 | 0.516 | 0.394 | 0.092 | -0.007 | -0.130 | -0.015 | 0.771 |
| CG | Total | -0.112 | 0.016 | 0.030 | 0.048 | - | 0.112 | 0.054 | -0.052 | 0.096 |
|  | Male | -0.105 | -0.001 | -0.248 | 0.025 | - | 0.021 | 0.119 | -0.010 | -0.200 |
|  | Female | -0.086 | 0.024 | 0.419 | 0.030 | - | 0.038 | -0.003 | -0.054 | 0.369 |
| CD | Total | -0.004 | -0.022 | 0.034 | 0.008 | 0.074 | 0.000 | 0.144 | 0.037 | 0.269 |
|  | Male | 0.022 | -0.017 | 0.129 | 0.033 | 0.045 | -0.009 | 0.008 | 0.133 | 0.343 |
|  | Female | -0.041 | -0.038 | 0.000 | -0.012 | 0.090 | 0.012 | 0.278 | -0.086 | 0.202 |
| CI | Total | -0.103 | 0.059 | 0.511 | 0.024 | 0.178 | -0.143 | -0.025 | 0.052 | 0.553 |
|  | Male | -0.073 | -0.016 | -0.555 | 0.179 | 0.145 | -0.054 | 0.377 | -0.028 | -0.025 |
|  | Female | -0.159 | 0.120 | 1.525 | -0.147 | 0.173 | 0.033 | -0.407 | -0.109 | 1.029 |
| ET | Total | -0.013 | -0.039 | 0.354 | 0.020 | 0.059 | 0.006 | 0.312 | 0.026 | 0.726 |
|  | Male | -0.010 | -0.010 | 0.185 | -0.026 | 0.227 | 0.005 | 0.140 | -0.015 | 0.495 |
|  | Female | -0.019 | -0.015 | 0.445 | 0.107 | -0.026 | -0.044 | 0.442 | 0.042 | 0.932 |
| GH | Total | -0.030 | -0.034 | -0.161 | 0.091 | 0.110 | -0.023 | 0.077 | 0.055 | 0.085 |
|  | Male | -0.036 | -0.022 | -0.038 | 0.090 | -0.019 | 0.001 | 0.026 | 0.022 | 0.022 |
|  | Female | -0.052 | -0.061 | -0.197 | 0.092 | 0.200 | -0.054 | 0.115 | 0.076 | 0.118 |
| GN | Total | -0.068 | -0.005 | -0.368 | 0.113 | 0.089 | 0.038 | 0.478 | -0.021 | 0.257 |
|  | Male | -0.025 | -0.060 | -0.239 | -0.032 | 0.162 | 0.054 | 0.150 | -0.031 | -0.021 |
|  | Female | -0.068 | -0.085 | -0.735 | 0.366 | 0.153 | 0.109 | 0.953 | -0.187 | 0.506 |
| KE | Total | -0.001 | -0.140 | 0.731 | -0.208 | 0.248 | -0.072 | -0.130 | 0.018 | 0.447 |
|  | Male | 0.079 | -0.061 | 0.225 | -0.323 | 0.041 | 0.045 | -0.510 | 0.838 | 0.334 |
|  | Female | 0.044 | -0.180 | 0.722 | -0.003 | 0.412 | -0.383 | 0.298 | 0.121 | 1.030 |
| LS | Total | 0.358 | -0.273 | 0.006 | -0.197 | 0.307 | -0.036 | 0.706 | -0.185 | 0.685 |
|  | Male | -0.090 | -0.244 | 0.451 | 0.074 | 0.243 | 0.057 | 0.074 | -0.140 | 0.425 |
|  | Female | 0.617 | -0.236 | -0.190 | -0.314 | 0.046 | 0.049 | 0.895 | -0.183 | 0.685 |
| LR | Total | -0.050 | 0.035 | -0.047 | 0.053 | 0.273 | -0.023 | 0.150 | -0.001 | 0.390 |
|  | Male | -0.066 | 0.107 | -0.012 | 0.028 | 0.054 | -0.027 | 0.249 | 0.006 | 0.340 |
|  | Female | 0.007 | -0.020 | -0.194 | 0.096 | 0.579 | 0.028 | 0.018 | -0.083 | 0.431 |
| MW | Total | -0.084 | -0.379 | 0.746 | 0.030 | 0.430 | -0.020 | 0.841 | -0.038 | 1.524 |
|  | Male | -0.068 | -0.217 | 0.409 | -0.102 | 0.321 | 0.047 | 0.648 | 0.024 | 1.063 |
|  | Female | -0.017 | -0.505 | 0.936 | 0.199 | 0.554 | -0.077 | 1.035 | -0.127 | 1.998 |
| ML | Total | -0.025 | 0.006 | 0.156 | -0.004 | 0.024 | 0.021 | 0.022 | -0.069 | 0.130 |
|  | Male | -0.028 | -0.011 | 0.369 | 0.025 | -0.013 | 0.030 | -0.070 | -0.098 | 0.205 |
|  | Female | -0.023 | 0.043 | -0.005 | -0.030 | 0.037 | -0.009 | 0.119 | -0.064 | 0.067 |
| MZ | Total | -0.102 | -0.076 | 1.388 | -0.132 | 0.728 | 0.078 | 0.326 | -0.117 | 2.094 |
|  | Male | -0.153 | -0.067 | 0.124 | -0.314 | 0.922 | 0.044 | 0.460 | 0.687 | 1.702 |
|  | Female | -0.030 | 0.082 | 1.672 | 0.009 | 0.564 | 0.114 | 0.244 | -0.226 | 2.428 |
| NE | Total | -0.020 | -0.011 | 0.016 | -0.025 | 0.025 | 0.052 | 0.131 | -0.006 | 0.162 |
|  | Male | -0.049 | 0.033 | 0.227 | -0.086 | 0.020 | 0.043 | 0.006 | -0.038 | 0.155 |
|  | Female | -0.011 | 0.013 | -0.098 | 0.026 | 0.018 | 0.034 | 0.175 | 0.011 | 0.168 |
| RW | Total | -0.048 | -0.119 | 0.066 | 0.029 | 0.164 | -0.011 | 0.303 | 0.012 | 0.394 |
|  | Male | -0.035 | -0.073 | -0.044 | -0.008 | 0.167 | 0.009 | 0.266 | -0.025 | 0.257 |
|  | Female | -0.023 | -0.126 | 0.180 | 0.076 | 0.156 | -0.084 | 0.311 | 0.059 | 0.549 |
| ST | Total | -0.023 | -0.034 | 0.129 | 0.000 | -0.042 | -0.020 | -0.069 | -0.039 | -0.098 |
|  | Male | -0.047 | -0.094 | 0.383 | -0.095 | -0.025 | 0.008 | -0.059 | -0.125 | -0.055 |
|  | Female | -0.015 | 0.067 | -0.116 | 0.077 | -0.072 | -0.032 | -0.091 | 0.045 | -0.136 |
| SN | Total | -0.007 | 0.027 | -0.133 | -0.046 | -0.025 | -0.040 | 0.094 | 0.009 | -0.121 |
|  | Male | -0.010 | -0.011 | -0.007 | -0.055 | -0.043 | 0.006 | -0.003 | 0.012 | -0.110 |
|  | Female | -0.003 | 0.091 | -0.218 | -0.032 | -0.010 | -0.132 | 0.170 | 0.007 | -0.125 |
| SL | Total | -0.033 | 0.043 | 0.373 | -0.056 | -0.018 | 0.025 | 0.084 | -0.043 | 0.373 |
|  | Male | -0.070 | 0.034 | 0.728 | -0.118 | -0.009 | 0.026 | -0.010 | -0.105 | 0.477 |
|  | Female | -0.017 | 0.069 | 0.106 | -0.015 | -0.054 | 0.044 | 0.148 | 0.002 | 0.282 |
| SZ | Total | 0.596 | -0.211 | -1.585 | -0.615 | 0.104 | 0.149 | 1.126 | 0.295 | -0.141 |
|  | Male | 1.037 | 0.282 | -1.844 | -0.154 | -0.090 | 0.146 | 0.817 | 0.025 | 0.219 |
|  | Female | 0.569 | -0.241 | -1.130 | -1.057 | 0.143 | -0.346 | 1.137 | 0.605 | -0.319 |
| TZ | Total | -0.042 | -0.079 | 0.258 | -0.085 | 0.066 | -0.084 | 0.523 | 0.021 | 0.578 |
|  | Male | -0.065 | -0.023 | 0.385 | -0.169 | -0.026 | -0.027 | 0.304 | 0.046 | 0.425 |
|  | Female | -0.026 | -0.105 | 0.161 | -0.006 | 0.127 | -0.152 | 0.690 | 0.001 | 0.690 |
| UG | Total | -0.075 | -0.156 | 0.330 | -0.154 | 0.150 | -0.019 | 0.209 | 0.086 | 0.370 |
|  | Male | -0.131 | -0.128 | 0.118 | -0.103 | 0.263 | 0.016 | 0.007 | 0.087 | 0.129 |
|  | Female | -0.046 | -0.103 | 0.585 | -0.266 | 0.086 | -0.089 | 0.330 | 0.057 | 0.553 |
| ZM | Total | -0.281 | -0.477 | 1.530 | 0.110 | 0.865 | -0.070 | 1.169 | -0.715 | 2.130 |
|  | Male | -0.301 | -0.677 | 0.953 | 0.246 | 0.952 | 0.071 | 0.677 | -0.390 | 1.531 |
|  | Female | -0.264 | -0.064 | 2.088 | -0.184 | 0.830 | -0.301 | 1.642 | -1.059 | 2.689 |
| ZW | Total | -0.159 | -0.127 | -0.971 | 0.157 | -0.049 | -0.218 | 0.927 | 0.183 | -0.256 |
|  | Male | -0.131 | -0.210 | -0.809 | 0.037 | -0.136 | -0.033 | 0.700 | 0.137 | -0.444 |
|  | Female | -0.193 | 0.379 | -1.037 | 0.229 | 0.004 | -0.689 | 1.083 | 0.190 | -0.032 |
